# Development of a genetic sensor that eliminates p53 deficient cells

**DOI:** 10.1038/s41467-017-01688-w

**Published:** 2017-11-13

**Authors:** Jovan Mircetic, Antje Dietrich, Maciej Paszkowski-Rogacz, Mechthild Krause, Frank Buchholz

**Affiliations:** 1 Medical Faculty and University Hospital Carl Gustav Carus, UCC Section Medical Systems Biology, TU Dresden, 01307 Dresden, Germany; 2 German Cancer Consortium (DKTK), OncoRay—National Center for Radiation Research in Oncology, Medical Faculty and University Hospital Carl Gustav Carus, TU Dresden, Dresden and German Cancer Research Center (DKFZ), 69120 Heidelberg, Germany; 3 Department of Radiation Oncology, Medical Faculty and University Hospital Carl Gustav Carus, TU Dresden, 01307 Dresden, Germany; 4 Helmholtz-Zentrum Dresden-Rossendorf, Institute of Radiooncology, 01328 Dresden, Germany; 5 German Cancer Research Center (DKFZ), Heidelberg and German Cancer Consortium (DKTK) Partner Site Dresden, 01307 Dresden, Germany; 6 National Center for Tumor Diseases (NCT), University Hospital Carl Gustav Carus, Technische Universität Dresden, 01307 Dresden, Germany; 7 Max Planck Institute of Molecular Cell Biology and Genetics, 01307 Dresden, Germany

## Abstract

The *TP53* gene fulfills a central role in protecting cells from genetic insult. Given this crucial role it might be surprising that p53 itself is not essential for cell survival. Indeed, *TP53* is the single most mutated gene across different cancer types. Thus, both a theoretical and a question of significant practical applicability arise: can cells be programmed to make *TP53* an essential gene? Here we present a genetic p53 sensor, in which the loss of p53 is coupled to the rise of *HSV-TK* expression. We show that the sensor can distinguish both p53 knockout and cells expressing a common *TP53* cancer mutation from otherwise isogenic *TP53* wild-type cells. Importantly, the system is sensitive enough to specifically target *TP53* loss-of-function cells with the HSV-TK pro-drug Ganciclovir both in vitro and in vivo. Our work opens new ways to programming cell intrinsic transformation protection systems that rely on endogenous components.

## Abstract (web summary)

TP53 is mutated in many cancers, a system to detect and selectively eliminate p53 mutant cells is an attractive therapeutic strategy. Here, the authors present a genetic sensor that can detect p53 activity and is coupled to the thymidine kinase gene, which can activate the drug Ganciclovir, resulting in cell death.

## Introduction

Because of its central role as a tumor suppressor protein in regulating a wide variety of stress signals and in preventing cellular transformation, p53 is commonly referred to as “the cellular gatekeeper” or the “guardian of the genome”^1,2^. Under normal physiological conditions, expression of p53 is kept at low intracellular levels^3^, but in response to cellular stresses such as DNA damage, oncogene activation, ribosomal stress and hypoxia, expression of p53 is rapidly induced and the protein is stabilized^4–6^. As a consequence, p53 exerts its function as a transcription factor, upregulating and downregulating genes implicated in cell cycle control, DNA repair, senescence and apoptosis^7–9^.

Considering p53’s central role as a guardian of the genome, it came as a surprise that mice deficient for p53 were developmentally normal^10^. One might have expected that such a vital cellular gatekeeper would be essential during embryogenesis, but it turned out that a normal p53 gene is not strictly required for mouse development. What is more, loss of p53 was clearly sufficient to predispose animals to many types of tumors^10^. Today, we know that around 50% of all human cancers show different types of *TP53* (gene encoding p53 in humans) alterations, making it the single most frequently mutated cancer-associated gene in the human genome^11,12^. Because of its prominent role in cancer, ways to target the p53 pathway have been long sought after. Although targeting transcription factors such as p53 remains challenging^13^, some progress in this regard has been described by the identification of drugs that activate or restore the function of p53 in cells that carry particular p53 mutations^14^. However, first-generation drugs have not yet shown hoped-for clinical responses^15^.

Advances in synthetic biology have made it possible to engineer cellular circuits with broad therapeutic potential^16,17^. Even though early efforts mostly focused on artificial gene networks in bacteria^18–20^, recent progress has shown that therapeutic synthetic networks can also be designed for mammalian cells^21,22^. However, the generation of genetic circuits that rely on endogenous eukaryotic proteins and that sense proteins at low abundance remain the exception^23–25^.

In this study, we build a genetic p53 device, capable of sensing the p53 status in human cells. Because the majority of all p53 alterations target the transcription factor function of the protein^26^, our sensor relies on detecting p53’s capacity to both activate and repress downstream genes. We show that the sensor is capable of discriminating p53 wild-type (WT) from otherwise isogenic, p53 knockout (KO) cells. Furthermore, the sensor can detect cells that express common p53 mutations, functions in primary cells and in an in vivo mouse model, suggesting that the sensor has widespread applicability in oncology research.

## Results

### p53 sensor design

**Fig. 1:**
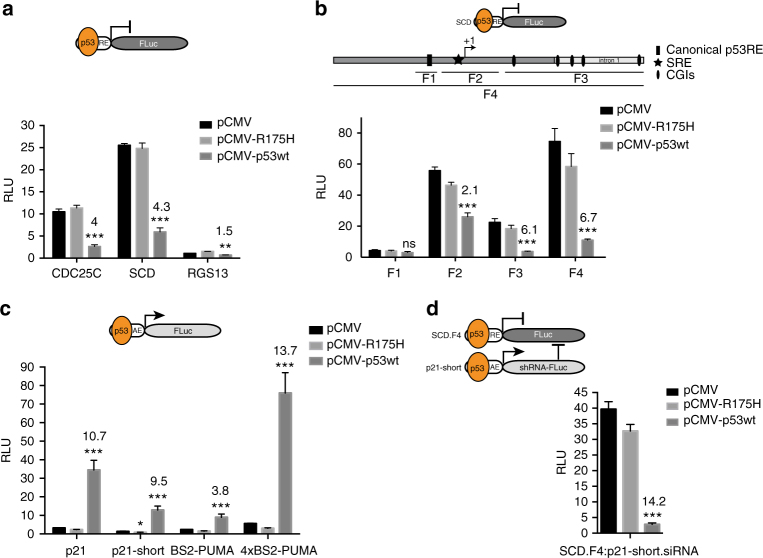
Development of a genetic p53 sensor. **a** Relative luciferase expression is shown for three p53-repressed elements (RE) derived from *CDC25C*, *SCD*, and *RGS13* promoters. **b** Relative luciferase expression is shown for four different fragments (F) derived from SCD promoter and/or 5′UTR/intron1. **c** Relative luciferase expression is shown for four p53-activated elements (AE) derived from p21 and PUMA promoters. **d** Combination of repressed and activated elements. Relative luciferase expression is given for cotransfection of indicated constructs. **a**, **b**, **c**, **d** The activation/repression folds were calculated between pCMV and pCMV-p53wt cotransfections and are given as numbers above the bars. All error bars represent the standard deviation (SD) of three independent experiments and Student’s two-tailed *t*-test values are represented comparing expression between pCMV and pCMV-p53wt cotransfections (***P < 0.001, **P < 0.01, *P < 0.05; ns, not significant; SRE, sterol regulatory element; CGIs, CpG islands)

In order to design a p53 sensor, we initiated investigation of a collection of different genetic elements derived from p53-regulated genes. To ensure broad-range sensitivity to p53 alterations, we opted to rely on elements from both p53-upregulated and p53-downregulated genes^27,28^. First, we commenced to sense p53 transcription repressing abilities. Three promoter elements from described p53-downregulated genes^29–32^ were tested in p53 KO HCT116 cells via coexpression of either the vector constitutively expressing WT p53 (pCMV-p53wt), a mutated version of the protein (pCMV-R175H) commonly found in a variety of tumors^26^, or the empty vector (pCMV). All three elements showed reduced luciferase expression when the cells were co-transfected with the plasmid encoding WT p53, whereas the mutant R175H-version and the control failed to repress luciferase expression, unmasking direct or indirect repressive activity of p53 on these promoters (Fig. 1a). A 1.1 kb element derived from the human *SCD* promoter displayed highest repression by WT p53 and also showed robust repression by WT p53 in RKO cells (Supplementary Fig. 1a), indicating that this effect is not cell line specific. Furthermore, the element successfully mediated enhanced p53 repression in p53 WT HCT116 cells in the presence of Nutlin-3^33^, signifying that drugs that stabilize p53 significantly increase the repression of this promoter element (Supplementary Fig. 1b). Recently, several regions outside of the *SCD* core promoter were also implicated in p53-mediated repression^34^. We systematically assessed repressive capabilities of all reported elements in the promoter, 5′UTR as well as in the first intron of the gene. A 1.8 kb element, that we term SCD.F4, showed around 60% increase in p53 suppression and was chosen for further development (Fig. 1b, Supplementary Fig. 1c).

Second, we investigated four promoter elements derived from *CDKN1A* (p21) and *PUMA*, two genes that are known to be activated by p53^35,36^. All elements showed robust induction of luciferase expression when p53 KO HCT116 cells were co-transfected with plasmids encoding WT p53, but not with the mutant R175H-version (Fig. 1c). An element of 100 bp derived from the p21 promoter (p21-short) showed strong luciferase activation, while the basal expression from this promoter was very low. Interestingly, this element was repressed by expression of the p53 R175H-mutant, indicating that this mutation switches the protein from an activator to a repressor on this model promoter (Fig. 1c). Based on these auspicious features the p21-short element was selected for further studies.

We then proceeded to combine repressed and activated elements in a two-gene network in which the luciferase output is co-repressed by WT p53. This was achieved through the use of the SCD.F4 element upstream of the luciferase gene in combination with the p21-short element driving the expression of a microRNA30a-imbedded short hairpin RNA (shRNA) targeting the luciferase transcript (Fig. 1d). When both elements were co-transfected together with the plasmid encoding WT p53, an enhanced effect in p53 suppression (from 6.7-fold for the SCD.F4 element alone to 14.2-fold for the two-gene sensor) of the output was observed (Fig. 1d), demonstrating the synergistic effect of these promoter elements in the network. Moreover, this effect was dose-dependent and even small amounts of p53 were sufficient to significantly suppress luciferase expression (Supplementary Fig. 1d).

### The sensor specifically targets p53 KO cells

**Fig. 2:**
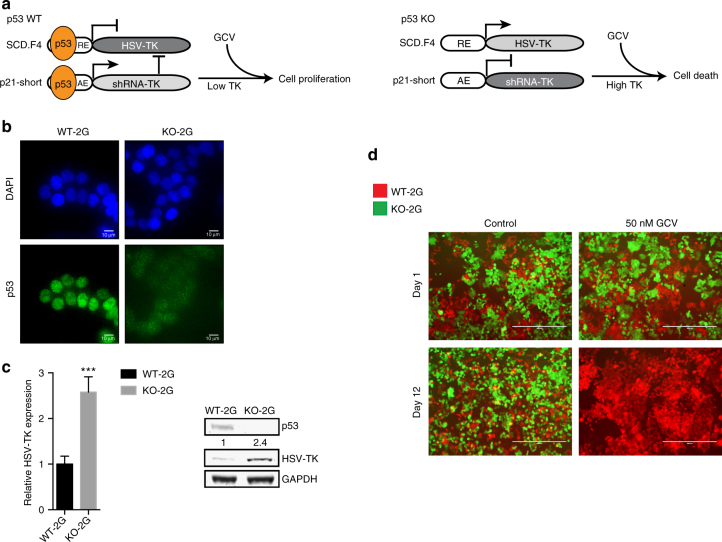
The 2G sensor can discriminate between p53 WT and KO cells and sensitizes KO cells to GCV. **a** Schematic outline of the 2G sensor with HSV-TK as a primary output and cellular outcomes after GCV treatment in p53 WT and KO cells. **b** Representative immunofluorescence (IF) images of DAPI (top panel) and p53 antibody-stained (bottom panel) WT-2G and KO-2G cells. Scale bars depict 10 μm. **c** Comparison of relative HSV-TK expression between WT-2G and KO-2G cells on either mRNA level by qPCR (left) or on protein level by Western blot (right). Error bars depict SD of three independent experiments and Student’s two-tailed *t*-test values are given (***P < 0.001). The relative quantification of the HSV-TK band signals is provided. **d** Representative images of the two-color assay in which a mix of WT-2G (mCherry-tagged) and KO-2G (GFP-tagged) cells was either treated with 50 nM GCV or control (water) for indicated period of time. Scale bars represent 400 μm

In order to address whether the sensor can function in the context of endogenous levels of p53, we exchanged the luciferase gene with *Herpes simplex virus* thymidine kinase (HSV-TK), to allow targeting of cells with the pro-drug Ganciclovir (GCV). Accordingly, the shRNA driven by the p21-short element was exchanged to target the HSV-TK transcript. Although not toxic per se, if provided with the drug GCV, HSV-TK phosphorylates the drug and induces DNA replication block followed by cell death^37,38^. We then integrated the sensor as a single construct (termed 2G, Supplementary Table 1) via piggyBAC transposition into p53 WT HCT116 cells (Fig. 2a). Low expression of HSV-TK was measured in tested clones, which was robustly increased after RNAi of p53, but not by RNAi of the closely related family member p73^39^ (Supplementary Fig. 2). This data suggested that the sensor is sensitive to endogenous p53 levels.

Subsequently, we established isogenic p53 KO cells directly from the p53 WT-2G clone, targeting the *TP53* gene utilizing CRISPR/Cas9. Several p53 KO clones were established, which all showed increased expression of HSV-TK when compared to the WT p53 parent cell line (Supplementary Fig. 3). One clone (KO-1, termed hereafter KO-2G, Supplementary Table 2) was chosen for further studies and displayed 2.4-fold higher expression of HSV-TK when compared to its WT p53 counterpart (Fig. 2b, c). To be able to directly compare sensitivity of WT-2G and KO-2G cells to GCV, the cells were subsequently tagged with mCherry (WT-2G) and GFP (KO-2G), respectively. In a two-color assay, both cell types were seeded together and treated either with 50 nM GCV or water as control. We observed rapid reduction of GFP-positive cells selectively in the GCV-treated samples and after 12 days could completely eliminate KO-2G cells, whereas the mCherry-tagged WT-2G cells proliferated in both conditions (Fig. 2d). Importantly, a 25 times lower concentration of GCV could still eliminate GFP-positive cells, albeit at a longer time interval, revealing a wide range of effective drug concentrations (Supplementary Fig. 4a). More detailed analyses of proliferation and cell survival revealed that GCV was severely impacting KO-2G cells without prominently affecting WT-2G cells (Supplementary Fig. 4b). These data demonstrate that p53 KO cells can be successfully targeted using the 2G sensor in vitro.

### The sensor distinguishes between WT and mutant p53

**Fig. 3:**
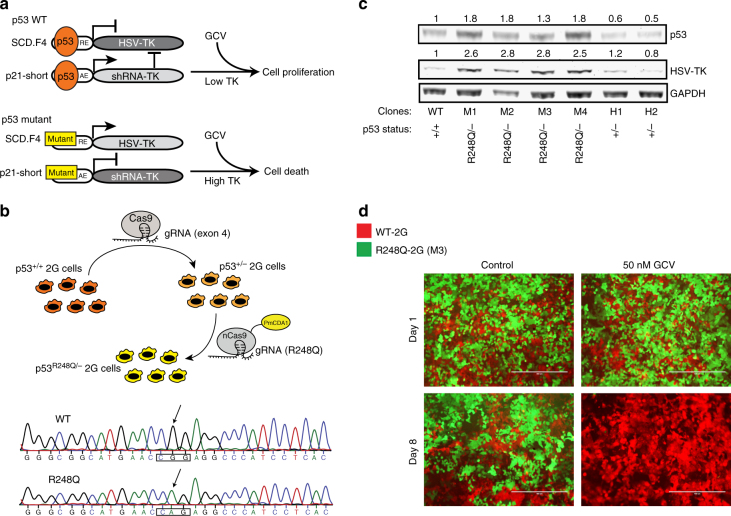
The sensor can discriminate between WT and a hot-spot mutant of p53. **a** Schematic representation of the effect of WT and mutant p53 on the sensor. **b** Top; illustration for generating hemizygous (p53^R248Q/−^) cells. Employed Cas9-proteins and the utilized gRNAs are shown. Bottom; electropherogram confirming the R248Q mutation in hemizygous (p53^R248Q/−^) cells. **c** Western blot depicting upregulation of HSV-TK in four different clones harboring the R248Q mutation. The relative quantification to GAPDH of HSV-TK and p53 band signals is shown. **d** Representative images of the two-color assay in which a mix of WT-2G (m-Cherry-tagged) and R248Q-2G mutant cells (M3 clone; GFP-tagged) was either treated with 50 nM GCV or control (water) for indicated period of time. Scale bars represent 400 μm

In addition to p53 deletions, many p53 alterations result in gain-of-function mutants that exhibit oncogenic properties^40,41^. To investigate whether the sensor can differentiate between mutant and WT p53 (Fig. 3a), we transiently overexpressed five different “hot spot” mutants in HCT116 p53 KO cells together with the sensor. Unlike WT p53 that robustly repressed HSV-TK expression, the tested mutants failed to efficiently repress HSV-TK (Supplementary Fig. 5a). In particular, the mutations V143A, R175H, and R249S, which are prominently found in tumor samples, were not effective in repressing HSV-TK expression. To investigate the sensor in a more physiological context, we transfected the sensor into primary human fibroblasts (WT p53), five cancer cell lines that carry different p53 mutations and a p53 negative cell line. Expression of HSV-TK was significantly higher in all cancer cell lines compared to the primary fibroblasts (between 4 and > 100 fold), indicating that the endogenously expressed p53 mutants cannot effectively repress HSV-TK expression (Supplementary Fig. 5b). We then proceeded to stably integrate one of the structural p53 mutants (R175H) into sensor-harboring KO-2G cells, mimicking the hemizygous status in most cancer types^42,43^. The resulting pool of cells was first tested for HSV-TK expression, which, consistent with our previous results, showed increased expression of HSV-TK compared to WT-2G cells (Supplementary Fig. 5c). To monitor if cells expressing the R175H mutant form can be specifically eliminated, the cells were subsequently tagged with GFP. Employing again the two-color assay, even low concentration of GCV preferentially targeted R175H cells (Supplementary Fig. 5d, e).

To test whether the sensor can detect an endogenously induced p53 mutation, we generated hemizygous R^248Q/−^ 2G clones (Fig. 3b), employing Cas9 base-editor technology^44^. Comparing HSV-TK protein expression of WT-2G cells with two heterozygous clones and four R248Q hemizygous clones, we found that while loss of p53 on only one allele did not influence HSV-TK expression, subsequent introduction of the R248Q mutation into the remaining WT allele led to a 3-fold increase in the sensor output (Fig. 3c). Consequently, R248Q mutant cells were efficiently eliminated upon GCV treatment and the cells were even more sensitive to GCV than p53 KO cells (Fig. 3d). Lower GCV concentrations eliminated R248Q cells as well, although longer incubation was required for complete depletion of mutant cells (Supplementary Fig. 6a, b). These data provide evidence of high sensitivity of the p53 sensor, whereby an endogenous single point mutation in the *TP53* allele could be sensed and used to successfully target mutant cells.

### The sensor is functional in primary cells

**Fig. 4:**
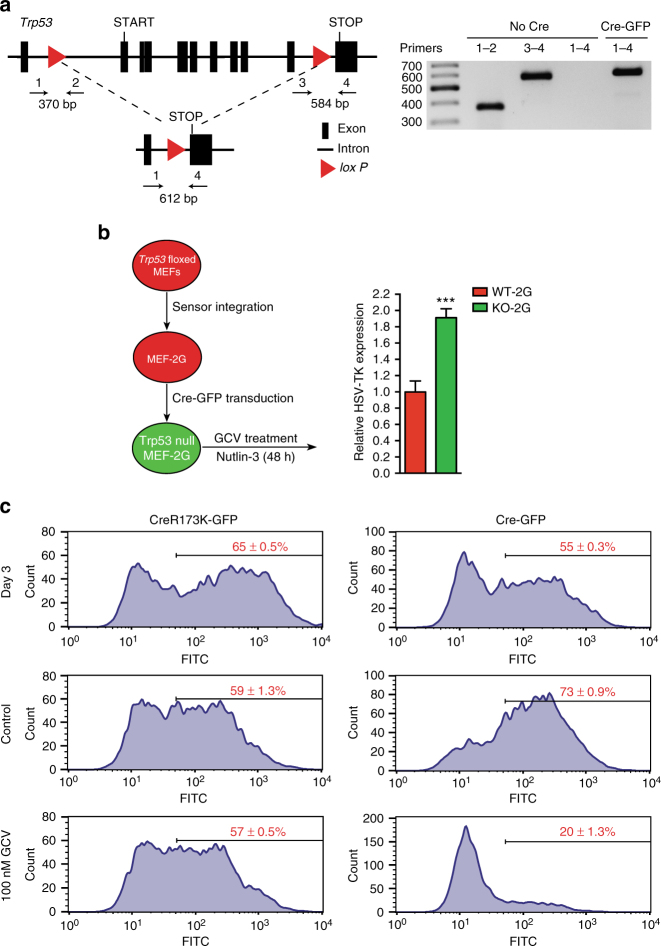
The sensor discriminates p53 WT from p53 KO cells in primary embryonic fibroblasts. **a** Left; schematic representation of the *floxed Trp53* allele in MEFs. Primers used for PCR are depicted. Right; agarose gel electrophoretogram depicts PCR products employing indicated primers in the presence (Cre-GFP) or absence (No Cre) of Cre recombinase. **b** Left; outline of the experiment in which the 2G sensor was integrated into *Trp53* floxed MEFs and then transduced with Cre-GFP expressing retrovirus (or an inactive version of Cre). The bar plot on the right shows relative HSV-TK expression in GFP-positive cells (p53 KO, green) and GFP-negative cells (p53 WT, red) in the presence of 5 μM Nutlin-3. All error bars represent SD of three independent experiments and Student’s two-tailed *t*-test values are given (***P < 0.001). **c** FACS histograms depicting the distribution of GFP intensity three days post transduction (top row), and after 6 days of additional control treatment (middle row) or GCV treatment (bottom row) for cells transduced with Cre virus (right) or the inactive recombinase (left). Mean percentages of GFP-positive cells of three replicates are shown

HCT116 and RKO are cancer cell lines that grow malignantly in the presence of WT p53. To investigate whether non-transformed, primary cells can also be sensitized to the loss of p53 we made use of mouse embryonic fibroblasts (MEF) carrying conditional *Trp53* alleles (*Trp53* floxed MEFs, Fig. 4a)^45^. After integrating the 2G sensor, we transduced these MEFs with GFP-bicistronic retroviral particles encoding either Cre or an inactive version of the recombinase (CreR173K)^46^ and confirmed that Cre transduction mediated effective *Trp53* deletion (Fig. 4a). As in the cancer cell lines, deletion of p53 resulted in increased expression of HSV-TK (Fig. 4b), in particular, when the cells were also treated with Nutlin-3 to stabilize p53 in the WT cells. Importantly, the *Trp53* floxed MEF-2G cells that had received Cre and thereby deleted *TP53* became sensitive to GCV treatment (Fig. 4c), while WT MEFs carrying the 2G sensor transduced with the Cre virus, *Trp53* floxed MEFs harboring no 2G sensor transduced with the Cre virus, or *Trp53* floxed MEFs carrying the 2G sensor transduced with the virus encoding the inactive Cre variant were all insensitive to GCV treatment (Fig. 4c; Supplementary Fig. 7). Taken together, these data revealed responsiveness of the sensor in primary cells.

### The sensor is functional in an in vivo model

We finally assessed whether the 2G sensor can specifically target p53 KO cells *i*n vivo. In order to address this question and directly compare the effect of GCV on WT-2G and KO-2G cells, both types were injected subcutaneously into opposite flanks of immunodeficient nude mice: WT-2G cells were injected into the right back side and KO-2G cells were injected into the left back side of the same animal. In parallel to tumor cell injection, mice were transplanted with an osmotic pump delivering either GCV (20 mg/kg bodyweight (BW)/day) or control solution (water).

**Fig. 5:**
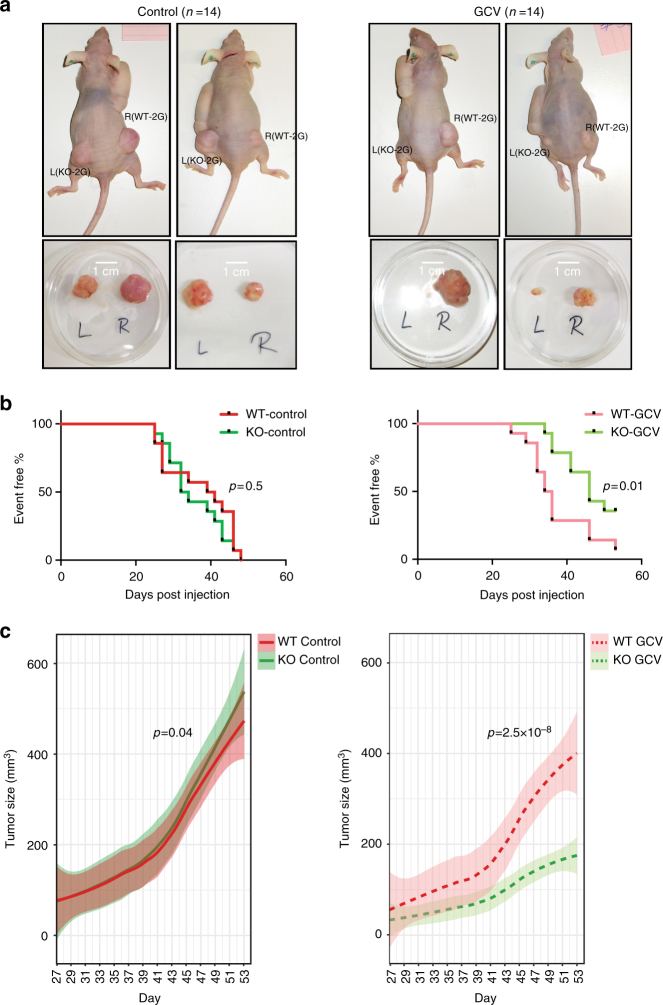
GCV specifically targets KO-2G tumors in vivo. **a** Top; representative in vivo images of WT-2G (right flank) and KO-2G (left flank) tumors 77 and 56 days post injection in control treatment group (water; left), and 86 and 82 days post injection in GCV-treated (right) mice. Bottom; ex vivo images of WT-2G and KO-2G tumors taken from the corresponding mice. **b** Kaplan–Meier graphs showing the proportion of mice without palpable tumors (set volume of 100 mm^3^) as a function of post-injection time (in days) for mice in control (left) and GCV-tretaed (right) groups. *p*-values comparing time to palpability between WT-2G and KO-2G tumors are shown. **c** Average tumor volume of WT-2G and KO-2G tumors is given as a function of post-injection time (in days) for control group of mice (*n* = 14, the left panel), and GCV treatment group (*n* = 14, the right panel). The curves show results of loess regressions of tumor sizes over time (span = 0.75) and the semi-transparent ribbons indicate 0.95 confidence intervals around the smooth. Indicated *p*-values are results of likelihood ratio tests comparing linear mixed effects models to respective nested models without an effect of cell type on tumor size

Fifty days post injection, WT-2G and KO-2G tumors had grown to similar sizes in the control group treated with water, demonstrating that both cell lines initiate tumors with similar growth kinetics as xenografts. In contrast, KO-2G tumors were much smaller than WT-2G tumors in mice treated with GCV (Fig. 5a). Time of palpability and growth kinetics of KO-2G tumors both confirmed their sensitivity to GCV treatment (Fig. 5b, c). Remarkably, GCV had no effect on WT-2G tumor formation, and only a minute effect on the tumor growth, while having a strong effect on both tumor formation and tumor growth in the case of KO-2G cells (Supplementary Fig. 8a, b). Hence, the 2G sensor was able to selectively eliminate only the cells lacking the *TP53* gene in an in vivo model. Taken together, the data from the in vivo experiments provide evidence that the difference in HSV-TK expression between p53 WT and KO cells opens a wide enough range for successful targeting by GCV.

## Discussion

Several recent advances in synthetic biology have focused on the design of mammalian transgene control devices that form a basis for the assembly of therapeutic networks in mammalian cells^16^. Though ever more complex genetic circuits have been developed, it has been challenging to construct modules relying on endogenous eukaryotic proteins. This is specifically true for mammalian transcription factors that usually represent a small fraction of the total proteome and where noise to signal ratio is thus quite high^47^.

With this work we present a genetic p53 sensor, capable of sensing both the absence of the WT protein and the presence of mutated versions of the protein. Our system demonstrates that endogenous mammalian proteins can be sensed, even at low levels. Built as a two-module unit, the sensor is a flexible construct, which should be amenable to further improvements. For instance, improving the scaffolding and targeting capabilities of the shRNA module could possibly contribute to future designs^48^. One important and interesting aspect would be to test if the sensor could be optimized toward particular p53 mutants, since it is known that transcriptional profiles of different gain-of-function mutants are not the same^9,40^. Finally, the overall strategy to build sensors should be applicable to other tumor suppressors. We envision that the combination of such sensors would allow the creation of robust transformation protection systems that could block cancer formation.

**Fig. 6:**
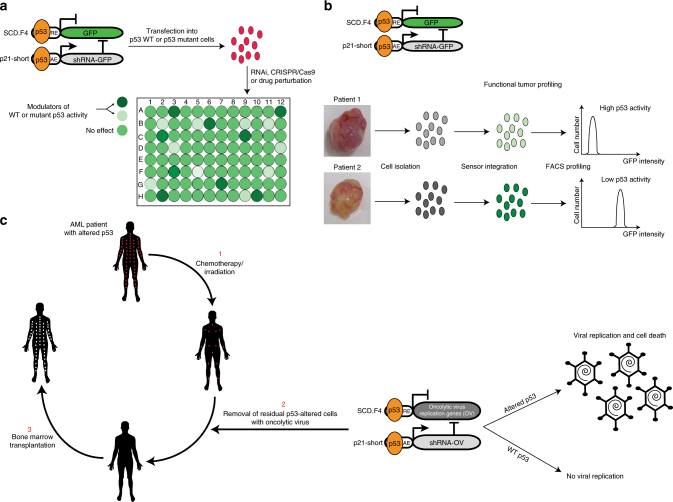
Possible p53 sensor applications. **a** Graphical illustration of the sensor’s use in large-scale drug or RNAi/CRISPR screens to uncover p53 stabilizers and/or drugs that restore WT p53 function in mutant cells. Important steps are highlighted by arrows. **b** Functional p53 profiling in primary tumor samples. A model to determine p53 activity in primary cancer cells is presented. **c** Clinical application of the sensor in AML patients with altered p53. An illustration to couple the sensor to oncolytic viruses and its application in a combination with radiotherapy/chemotherapy before bone marrow transplantation is presented. Red and white dots illustrate leukemic p53 mutant cells and healthy donor cells after transplantation in the patient, respectively. Arrows highlight important steps

The modular sensing device presented here can be seen as an illustration of a rational synthetic biology approach to both interrogating and programming cellular function. Whether the described network acts as a sensor only or as a programming device is determined solely by the primary output and can be accordingly adjusted. In the case of GFP or any other easily detectable marker, the sensor functions as a reporter to gauge activity of the endogenous protein. This feature of the sensor can be particularly useful in designing screens for probing modulators of p53 activity. For example, drug screens or genome wide RNAi/CRISPR screens could reveal stabilizers of WT p53 or drugs that revert mutant p53 back to WT function^49^ (Fig. 6a). Since both of these events would result in reduction of primary output signal, one could either select for specific cells (HSV-TK as a primary output) or simply monitor the signal (GFP as a primary output). In addition, many novel approaches, including synthetic lethality and collateral vulnerability screens, are being developed to either target defects in p53 or elucidate further the function of the protein^50,51^. Here too, we suspect, the sensor could serve a role.

We also foresee that the p53 reporter could be usefully employed in clinical applications. Sequencing the p53 gene in tumor samples provides the mutation status of the gene, but this information says little about the specific p53 activity in the tumor cells due to dominant negative protein interactions, mislocalization or MDM2 overexpression^52–54^. Transferring the sensor into primary tumor cells followed by readout of the output would provide a measure to estimate p53 activity in the cells (Fig. 6b). This information could contain prognostic and/or diagnostic value.

Finally, we envision possible therapeutic potential for a genetic p53 sensor. Coupling the p53 status to cell survival outcome provides an opportunity to target cells with compromised p53 function. The example of HSV-TK and GCV we provide in the study is only one such possibility. Different enzyme/prodrug systems could be used too in order to circumvent the problem of drug resistance^55^. Important hurdles, however, would need to be considered if the sensor would be used clinically. First, as is the case for any gene therapy approach, efficient and safe delivery of a sensor would need to be resolved. Recent advances in the field of oncolytic viruses provide a particularly appealing route to dealing with this problem. Several oncolytic viruses have been either in clinical trials or already approved for therapeutic purposes^56^, and it has been shown that prodrug-activating enzymes armed oncolytic viruses promote synergy with prodrugs^57^. Alternatively, the sensor could be used to modify oncolytic viruses so that they are capable of replication only in p53-deficient cells. This could be accomplished by introducing genes necessary for viral replication as the primary output of the sensor (Fig. 6c). Clinical application of this approach can be illustrated by the example of acute myeloid leukemia with complex karyotype (CK-AML) where the alteration of WT p53 is known to lead to exceedingly dismal outcomes^58,59^. In these patients standard chemotherapy/irradiation treatment to eradicate the tumor cells before transplantation is typically not sufficient to eliminate all malignant cells. As a consequence, rapid relapse is frequently observed in patients receiving allogeneic bone marrow transplants. Oncolytic viral particles that specifically destroy p53-altered cells might overcome this shortcoming and remove all mutated cells that remain after patient irradiation/chemotherapy (Fig. 6c). All of the mentioned therapeutic possibilities face, however, the problem of variable p53 expression levels in different cell types and tissues. Whether the physiological p53 levels are high enough to suppress the sensor output in p53 WT cells would need to be addressed for each case separately. One initial step in this direction would be to generate a transgenic mouse that carries the sensor and then investigate the sensor output (HSV-TK) in different tissues, providing essential information about biosafety. Overall, our work opens new ways for thinking about programming cells by designing genetic safeguards that protect organisms from cancer development.

## Methods

### Cell culture and transfection

HCT116 and RKO (*TP53* WT and KO) cells (a kind gift from B. Vogelstein, Sidney Kimmel Comprehensive Cancer Center, Johns Hopkins University, Baltimore, USA) as well as MEFs (WT and *Trp53* floxed, a kind gift from A. Berns, the Netherlands Cancer Institute, Amsterdam, The Netherlands and Skoltech Center for Stem Cell Research, Skolkovo, Russia), HEK293, LS123, WiDr and IMR90 cells (all acquired from ATCC; HEK293—CRL3216, LS123—CCL255, WiDr—CCL218, IMR90—CCL186) were cultivated in 4.5 g/l glucose Dulbecco’s Modified Eagle’s Medium (DMEM) supplemented with 10% fetal bovine serum (FBS), 100 U/ml penicillin and 100 μg/ml streptomycin (Gibco). BT-549 and COLO320DM (acquired from ATCC; BT-549—HTB122 and COLO320DM—CCL220) were cultured in ATCC-formulated RPMI-1640 Medium, supplemented with 10% FBS, 100 U/ml penicillin, and 100 μg/ml streptomycin. All the cell types were cultured at 37 ^o^C and 5% CO_2_. WiDr cells are the only one on either ICLAC or NCBI list of commonly misidentified cells, but the misidentification stems from confusion with HT-29, also p53 R273H mutant cell line, thus not effecting conclusions of the experiment (Supplementary Fig. 5b). All cells were checked for mycoplasma contamination and none was positive.

All the plasmid constructs were transfected using Lipofectamine 2000 (ThermoFisher) reagent except for the CRISPR/Cas9 constructs in HCT116 (see Establishment of p53 heterozygous, KO and mutant cells) and IMR90 cells that were transfected using Amaxa Cell Line Nucleofector Kit V and R, respectively (Lonza), following the manufacturer’s recommendations. siRNAs (esiRNA, Eupheria Biotech) were transfected using Oligofectamine (ThermoFisher). In 96-well plate format, 200 ng plasmid DNA was mixed with 25 μl Opti-MEM media (Gibco, ThermoFisher) and 0.25 μl Lipofectamine 2000 reagent in 25 μl Opti-MEM media was then added. For RNAi experiments, 100 ng siRNAs in 25 μl Opti-MEM media was mixed with 1 μl Oligofectamine in 25 μl Opti-MEM. For all the other plate formats, the amount of plasmid DNA or siRNA was scaled according to the surface area of the wells. Other constructs used in the study are: pCMV (addgene #16440), pCMV-p53wt (addgene #16434), pCMV-V143A (addgene #16435), pCMV-R175H (addgene #16436), pCMV-R248W (addgene #16437), pCMV-R249S (addgene #16438), pCMV-R273H (addgene #16439), pSpCas9-2A-GFP (PX458, addgene #48138), and nCas9-PmCDA1 (addgene #79620).

Stable HCT116 p53 WT as well as *Trp53* floxed MEF cells expressing the 2G sensor were established upon sensor integration via transposition, done by transfecting the 2G plasmid together with a transposase expressing vector (System Biosciences, #PB210PA-1) in a 2.5:1 ratio and subsequent puromycin selection (2 μg/ml, Puromycin Dihydrochloride, ThermoFisher). Fluorescent tagging of the 2G clones (WT and KO) was done by stably integrating plasmids expressing mCherry and GFP, respectively. The pCAGGS-*loxP*-Cherry-*loxP*-EGFP vector (a kind gift from Elly Tanaka) from which EGFP sequence was removed via BamHI sites, and GFP-expressing pfwB plasmids (addgene #37329) were used. In the case of pwfB, cells were selected with blasticidin (ThermoFisher, 10 μg/ml), while cells transfected with mCherry-expressing plasmid were not selected, but sorted for mCherry twice, 7 and 21 days post transfection. For establishing p53 mutant-expressing cells, pCMV-neo-Bam pasmids were used (addgene #16435, #16436, #16437, #16438, and #16439). Cells were selected in 400 μg/ml geneticin-containing media.

### Design of sensor elements

Both p53 repressed and activated elements were first PCR-amplified from human genomic DNA (HCT116 cells) and then cloned into pGL3-Basic luciferase vector (Promega) via KpnI and either NheI or XhoI restriction sites, using primers given in Supplementary Table 3, except for the 4xBS2-PUMA element which was cloned directly using the oligonucleotides also given in the table. To construct *SCD* intronic region (fragments F3 and F4, see Fig. 1b), we first removed seven ATG codons from the intron (fragment was synthesized by GeneArt, ThermoFisher) and then moved this element into the large SCD fragment via BlpI and XhoI sites (Supplemetary Table 3). This new fragment (F4) then served as a tamplate for F3 element.

All miR30a-embedded shRNAs (targeting firefly luciferase and GFP) were amplified in several steps using the *miR30a* gene as a starting target DNA. In all cases, embedding was done via overlapping PCR reactions. The first reaction amplified the 5′ end of *miR30a* up until the start of the shRNA stem-loop and used miR30a forward primers. The reverse primer in this reaction contained a 20 bp overhang complementary to first 20 bp of 5′ forward primer used for the second PCR reaction (shRNA reverse primer, see Supplementary Table 3). The forward primer used in the second PCR reaction contained the whole shRNA stem-loop for targeting FLuc, GFP, and HSV-TK (shRNA forward primer, see Supplementary Table 3) and the reverse primer annealed to the 3′ end of *miR30a* (miR30a reverse primer). In this way after the two PCR reactions, two overlapping PCR products were created, that were then used in the third PCR reaction that amplified the whole transducer module. These shRNAs were then cloned together with p21-short element into the piggyBAC plasmid (System Biosciences, #PB510B-1). Since none of the tested shRNAs against HSV-TK gene achieved substantial knock-down, in the final 2G targeting plasmid shRNA against GFP was used, and a sequence recognized by the shRNA was introduced into the HSV-TK 3′ untranslated region as a triple tandem repeat via FseI and XbaI sites (the complete sequence of the 2G vector is given in the Supplementary Table 1).

HSV-TK coding region was amplified from pAL119-TK plasmid (addgene #21911) using primers listed in Supplementary Table 3.

### Luciferase assay

In all luciferase experiments, constructs expressing FLuc were mixed with constructs expressing either WT p53, mutated p53 or the empty vector in a 10:1 ratio and with Renilla luciferase-expressing vector (pRL-CMV, Promega) in a 100:1 ratio. In the experiment where the SCD.F4-driving luciferase construct was cotransfected with p21-short-driving shRNA plasmid, the ratio used was 20:1. Luciferase luminescence was measured 48 h post cotransfection using Dual-Glo Luciferase Assay System (Promega) on the EnVision Multilabel Reader (Perkin Elmer). Luciferase signal was normalized to signal coming from the empty luciferase vector (pGL3) and Renilla luciferase. The average values and standard deviations of three independent experiments are presented.

### Establishment of p53 heterozygous, KO, and mutant cells

p53 WT-2G cells were transfected with Cas9/gRNA expression vector (PX458) using Amaxa Cell Line Nucleofector Kit V (Lonza) following the manufacturer’s recommendations. In the case of obtaining a KO clone, three different gRNAs were used (Supplementary Table 4), while in the case of a heterozygous clone only gRNA2 was used. gRNAs were cloned as annealed oligos using SsbI restriction enzyme. DNA isolated from transfected cells was tested using the T7 endonuclease surveyor assay. Cells were then sorted as single cell clones using the BD FACSAria III sorter (BD Biosciences) in 96-well plate and after IF staining for p53, four p53 KO clones were tested for HSV-TK expression (Supplementary Fig. 3) and the clone number 1 (KO-1) was chosen for further experiments. To confirm the KO status of *TP53* in this clone, the regions around the gRNA-induced cuts were PCR-amplified using primers listed in Supplementary Table 5 and cloned using the TA cloning Kit (ThermoFisher) into pCR2.1. DNA from 20 different bacterial colonies were analyzed by sequencing. The same procedure was used to confirm the status of heterozygous clone. Sequences of the clone KO-1 and the p53 heterozygous clone from which R248Q mutant was derived are shown in Supplementary Table 2.

In order to establish p53 R248Q mutant cells, p53 heterozygous cells were electroporated twice with an nCas9-PmCDA1-expression construct also expressing the R248-targeting gRNA (see primers listed in Supplementary Table 4) using the Amaxa Cell Line Nucleofector Kit V (Lonza). Four days post electroporation, cells were single-cell sorted into 96-well plates. DNA was isolated from 24 clones and the region around R248 was PCR-amplified and sequenced using primers given in Supplementary Table 5.

### IF staining of Cas9/gRNA-transfected WT-2G cells

Sorted cell clones were fixed in 96-well plates using 4% formaldehyde (Sigma F8775) in 1× PBS for 15 min at 22 ^o^C. After two brief washes with 1× PBS + 30 mM glycine, cells were permeabilized with 1× PBS + 0.5% Triton X-100 for 5 min at 4 ^o^C, followed by again two washes with 1× PBS + 30 mM glycine. Afterwards cells were blocked in blocking solution (1× PBS, 0.2% fish skin gelatin) for 15 min at room temperature (RT). Primary antibody was diluted in blocking solution and incubated for 1 h at RT (mouse anti-p53, DO-1, Santa Cruz Biotechnology, 1:800). Cells were washed three times for 3 min each in blocking solution followed by a 30 min incubation at RT with secondary antibody, which was diluted in blocking solution. As secondary antibody fluorescently labeled donkey anti mouse-IgG antibody (AlexaFluor 488, ThermoFisher, 1:500) was used. After three final washes for 3 min each in blocking solution, plates were incubated for 5 min with blocking solution containing 1 μg/ml DAPI, followed by one wash in blocking solution and finally covered with PBS.

Images were acquired with a DeltaVision Core Microscope (Applied Precision, Olympus IX71 microscope) using a 60× /1.42 UplanSApo oil-immersion objective. Z-stacks (0.2–0.5 μm optical sections) were collected and deconvolved using softWoRx (Applied Precision).

### Quantitative PCR

RNA was extracted using the RNeasy mini Kit (Qiagen) according to manufacturer’s instructions (in the case of RNAi knock-downs, 48 h post transfection). To eliminate genomic DNA contamination, an on-column DNase I digestion step was included in the extraction process. The concentration and quality of RNA was measured using NanoDrop 1000 Spectrophotometer (Thermo Scientific) by measuring the absorbance at 260 nm.

For cDNA synthesis, 1 μg of RNA was reverse transcribed using SuperScript III first strand synthesis Kit (ThermoFisher) with Oligo dT_12–18_ (ThermoFisher) primers. In the qPCR reaction, 70 nM primers were used (sequences and annealing temperatures for all the genes given in the Supplementary Table 6) together with Absolute qPCR SYBR green mix (Thermo Scientific). Reaction were carried out on a CFX96 Touch Real-Time PCR Detection System (Bio-Rad). A single PCR product was confirmed by analyzing the melting curve of the qPCR reaction for a single peak. Wherever possible, the qPCR primers were designed so as to span exon–exon junctions and primers were analyzed for efficiency by testing the performance on a dilution series. Target mRNA levels were normalized against the levels of TATA-binding protein or Glyceraldehyde 3-phosphate dehydrogenase (GAPDH) transcripts and the ∆∆Ct method was used for relative quantification of the mRNA levels of the genes of interest. When HSV-TK expression was measured and compared between different cell lines (Supplementary Fig. 5b), GFP-expressing plasmid pfwB was cotransfected and used for normalization using primers listed in Supplementary Table 6.

Statistical analysis was done using Student’s two-tailed *t*-test.

### Western blot

Samples were boiled in Laemmli buffer (Sigma Aldrich) and subjected to SDS–PAGE (NuPage 4–12% Bis-Tris gels; ThermoFisher). Gels were blotted to nitrocellulose (Protran; Schleicher & Schuell), blocked in 5% nonfat milk in PBST (PBS containing 0.1% Tween-20) for 1 h at RT and incubated over night at 4 ^o^C with primary antibody. The primary antibodies used were: goat anti-HSV-TK (Santa Cruz Biotechnology, sc-28037, 1:500 dilution), rabbit anti-GAPDH (abcam, ab9485, 1:8000 dilution), and mouse anti-p53 (Santa Cruz Biotechnology, sc-126, DO-1, 1:1000 dilution). The next day, membranes were washed three times for 10 min each in 5% milk PBST and were then incubated for 45 min at RT with secondary antibodies (donkey anti-goat IRDye 800CW and donkey anti-mouse or donkey anti-rabbit IRDye 680LT, all LI-COR Odyssey, 1:14 000). Membranes were washed three times for 10 min each in PBST followed by one PBS wash. Bands were visualized with the LI-COR Odyssey imaging system. As a molecular weight standard, Spectra Multicolor Broad Range Protein Ladder (Fermentas) and MagicMark XP Western Protein Standard (ThermoFisher) were used. Uncropped versions of all western blots used in the study are presented in Supplementary Fig. 9.

### Two-color assay

For the two-color assay, WT-2G (mCherry-tagged) and either KO-2G (GFP-tagged), KO-2G-R175H (GFP-tagged) or R248Q (GFP-tagged) cells were seeded together (50,000 cells each) and treated either with water or 50 nM GCV (Sigma, G2536). Treatment lasted for 5 days after which cells were incubated for 5 days without GCV. Cells were split after 2 days and 100,000 cells were seeded in a new dish. In the case of low GCV treatment, 50,000 WT-2G and 50,000 mutant cells (KO-2G, R175H or R248Q) were seeded into 12-well plates and treated with control (water) or 2 nM GCV. Cells were split every third day without removing the drug. In all two-color assays, cells were imaged with an EVOS FL Cell Imaging System (ThermoFisher) using 10× objective and by superimposing images obtained from RFP and GFP channels.

In order to assess the effect of GCV separately on WT-2G, KO-2G, R175H, and R248Q cells, all four cell types were seeded in 12-well plates (80,000 cells) and treated with 1 nM GCV. Cells were split every third day without removing the drug. On the day of splitting, cell numbers were determined using the Countess II Fl Automated cell counter (ThermoFisher; dead cells were discriminated using Trypan Blue stain, ThermoFisher). Cell numbers of GCV-treated wells were normalized to those of control-treated (water) wells.

### Transduction of the Cre-expressing virus

Viral Cre recombinase expression constructs (Cre WT and Cre R173K) were based on pBabe-puro plasmid containing an internal ribosomal entry site and GFP reporter gene (Addgene plasmid #1764). Cre WT and Cre R173K coding sequences were introduced into pBabe-puro plasmid as described previosuly^60^. Virus was produced in HEK293T cells. Cells (3,000,000) were transfected with 18 μg pBabe-puro constructs containing one of the two recombinases, 5 μg plasmid encoding ecotropic envelope and 10 μg plasmid encoding gag-pol proteins, using Lipofectamine 2000 (ThermoFisher). 24 h after transfection, medium was exchanged to low glucose DMEM medium and cells were incubated for 24 h in this medium at 32 °C after which viral supernatants were collected. For infection, *Trp53* floxed MEF cells expressing the 2G sensor (or *Trp53* floxed MEF cells without the sensor and WT-2G MEFs, 40,000 cells) were plated into 12-well plates. 24 h later the cells were treated with 2 ml retroviral supernatant in the presence of 4 μg/ml polybrene and 10 mM Hepes, spun down for 30 min at 2500 rpm and incubated for 7 h at 37 °C before fresh medium was added. To confirm that Cre transduction was resulting in deletion of a portion of *Trp53* alleles, GFP-positive and GFP-negative cells were sorted and genomic DNA was isolated from them. PCR confirmation of *Trp53* deletion was performed using primers listed in Supplementary Table 5. The uncropped version of the gel is presented in Supplementary Fig. 9.

### Targeting the *Trp53* floxed MEFs

Three days after transduction, without separating GFP-positive from GFP-negative cells, *Trp53* floxed MEF-2G cells (or WT-2G and *Trp53* floxed MEFs) were split in the presence of 5 μM Nutlin-3 and 1 day later treated with either 100 nM GCV or water. GCV treatment lasted for 6 days, after which the percentage of GFP-positive cells was measured on a FACSCalibur cytometer (BD Biosciences) using CellQuest Pro software. All the samples were analyzed in three replicates.

### Targeting the KO-2G tumor xenografts in mice

NMRI (nu/nu) mice (12-weeks-old, male and female in equal numbers and of equal weight) were obtained from the pathogen-free animal breeding facility at OncoRay-National Center for Radiation Research in Oncology, Faculty of Medicine and University Hospital Carl Gustav Carus, Technische Universität Dresden. The animal facility and the experiments were approved according to Faculty of Medicine and University Hospital Carl Gustav Carus’s guidelines and German animal welfare regulations. Mice were fed with commercial laboratory animal diet and water ad libitum.

WT-2G and KO-2G cells were used for subcutaneous transplantation and implantable osmotic pumps (Alzet pumps, model 2002, DURECT Corporation) were used for continuous delivery of GCV (Cymevene infusion vials, Roche, diluted in water) over 14 days. GCV administered concentration was 20 mg/day/kg BW. For further immunosuppression, nude mice were total-body irradiated 3 days before tumor inoculation (4 Gy, 200 kV X-rays, 0.5 mm Cu filter, 1 Gy/min). Prior to transplantation, mice were anesthetized via intraperitoneal injection of ketamine (120 mg/kg BW, Sigma-Aldrich) and xylazine (16 mg/kg BW, Sigma-Aldrich). Pumps were transplanted according to the manufacturer’s recommendations. In brief, the skin between scapulae was incised and a subcutaneous pocket was created for pump implantation. The procedure was directly followed by subcutaneous tumor cell injection at the flanks of the mice (WT-2G cells at the right side and KO-2G cells at the left side, respectively). Single cell suspensions were prepared in 1× PBS and mixed with Matrigel Matrix (BD Biosciences) at a 1:1 (v/v) ratio. A total volume of 50 µl containing 10,000 tumor cells was used for injection with insulin syringes. Animals were not randomized. However, to avoid that animals of different groups were injected with a time shift (which could influence the viability of cells in suspension), the injected animals were allocated to alternating groups after transplantation. Tumor formation was controlled every second day and growth was monitored two times a week by determining the tumor axes *x* (longest tumor axis) and *y* (perpendicular axis) using a manual caliper. Tumor volumes were estimated by the formula of a rotational ellipsoid: *π*/6 × *x* × *y* ^2^. Observer blinding was conducted. Animals were sacrificed when the first tumor reached a diameter of 20 mm or when the animal appeared to suffer. The maximum observation period was 90 days.

The proportions of event free injection sites were interpreted as Kaplan–Meier estimators and the event was defined as tumor volume exceeding 100 mm^3^. This was chosen because palpability of smaller nodules was difficult to monitor and thus was not stringently evaluable. The resulting Kaplan–Meier graphs (Fig. 5b) show the proportion of injection sites where no growing tumor was detected as a function of time after injection. Two-sided log-rank tests were used to compare tumor incidence between different groups. Sample sizes for the Logrank-Test were estimated based on data of the wt HCT116^61,62^. Assuming that on day 12 after tumor cell injection 80% of tumors in the control group and 30% of tumors in the experimental group were palpable and using *α* = 0.05 and *β* = 0.2, a sample size of 15 animals was calculated. One animal was added to account for potential drop out. Due to infections at the pump transplantation site two animals per group were sacrificed early after transplantation and were not included in the analysis.

To compare tumor volumes in mice treated with GCV and control mice, we used R to perform two two-way analyses of variance of KO and WT tumors separately. As dependent variables we used natural logarithms of tumor volumes plus one, and as independent variables we used time and treatment, without interaction. Significance of the treatment effects was assessed using *F*-test. To analyze differences between WT and KO tumors in treated and non-treated mice, we used R package lme4 [doi:10.18637/jss.v067.i01] and performed a linear mixed effects analysis of the relationship between a natural logarithm of tumor size plus one and cell type (in GCV and non-treated mice independently). As fixed effect, we entered time and cell type (without interaction term) into the models. As random effects, we had intercepts for mice, as well as by-mouse random slopes for the effect of time. *P*-values associated with effect of cell types were obtained by likelihood ratio test of full models against models without the effect in question.

Unexpectedly, sequencing of the remaining tumors in GCV-treated group of mice revealed that the left flank tumors (injected as KO-2G cells) were p53 WT. Indeed, molecular inspection uncovered that the cells isolated from the left side were WT-2G cells. Hence, KO-2G cells were either contaminated with WT-2G cells at the time of injection, or some WT-2G cells had migrated to the injection point of the KO-2G cells. In any event, these results indicate that the presented results underestimate the efficiency by which KO-2G cells were eliminated in the mouse model.

### Data availability

All relevant data are available from the authors.

## Electronic supplementary material

Supplementary Information
