# Supplementary material for: Development of a genetic sensor that eliminates p53 deficient cells
Source: Nat Commun. 2017 Nov 13;8:1463. doi: 10.1038/s41467-017-01688-w (PMC5684360; doi:10.1038/s41467-017-01688-w)
Supplement: Supplementary file 1 — Supplementary Information [file 41467_2017_1688_MOESM1_ESM.pdf]

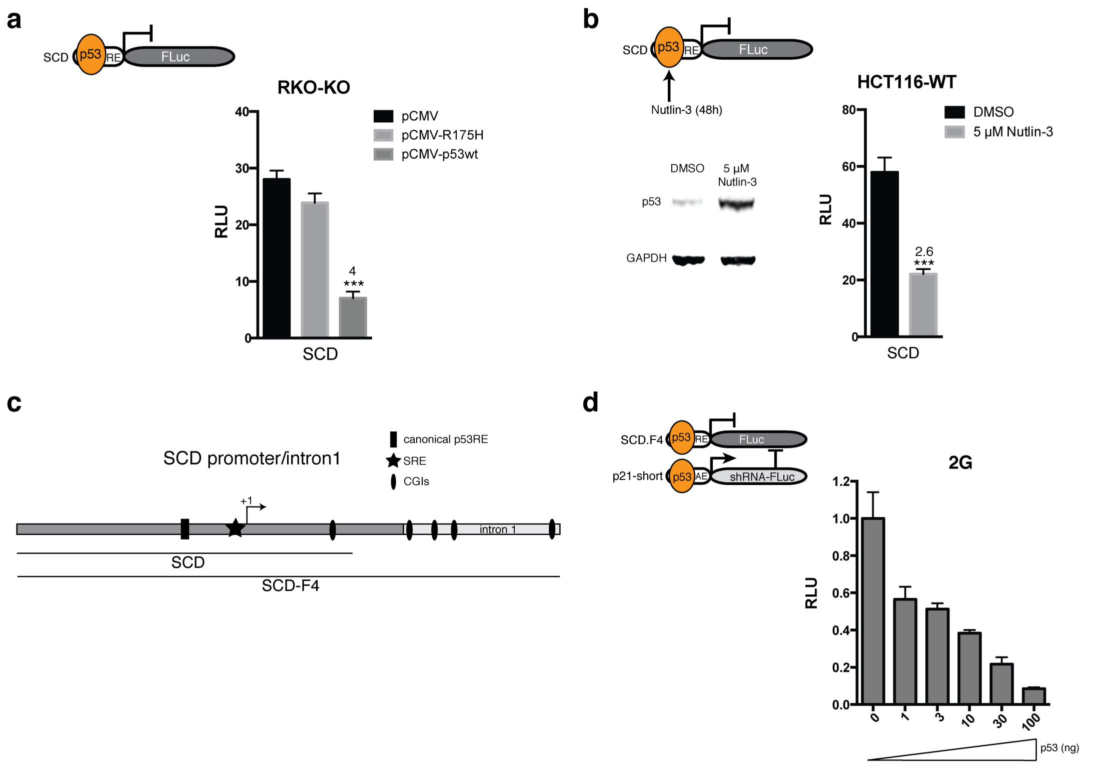

Supplementary figure 1: An element derived from *SCD* gene is robustly repressed by p53. (**a**) Relative luciferase expression is shown for the SCD element in RKO p53 KO cells. The repression fold was calculated between pCMV and pCMV-p53wt cotransfections and is given as number above the bar. (**b**) Left; western blot depicts increase in p53 levels upon Nutlin-3 treatment of HCT116 p53 WT cells. Right; luciferase response of the SCD element in HCT116 p53 WT cells upon treatment with Nutlin-3. The suppression fold is given as number above the bar. (**c**) Schematic representation of *SCD* promoter and 5'UTR/intron1. Comparison between SCD and SCD.F4 fragments is shown (RE – repressed element; SRE – sterol regulatory element; CGIs – CpG islands). (**d**) Luciferase response of the SCD.F4 element in combination with p21.short-shRNA targeting luciferase is dose-dependently suppressed by p53 in HCT116 p53 KO cells. (**a**, **b** and **d**) Error bars represent standard deviation (SD) of 3 independent experiments and Student's two-tailed t-test values are given comparing expression between pCMV and pCMV-p53wt cotransfections (in **a**) or Nutlin-3 and DMSO treatment (in **b**, *** P<0.001).

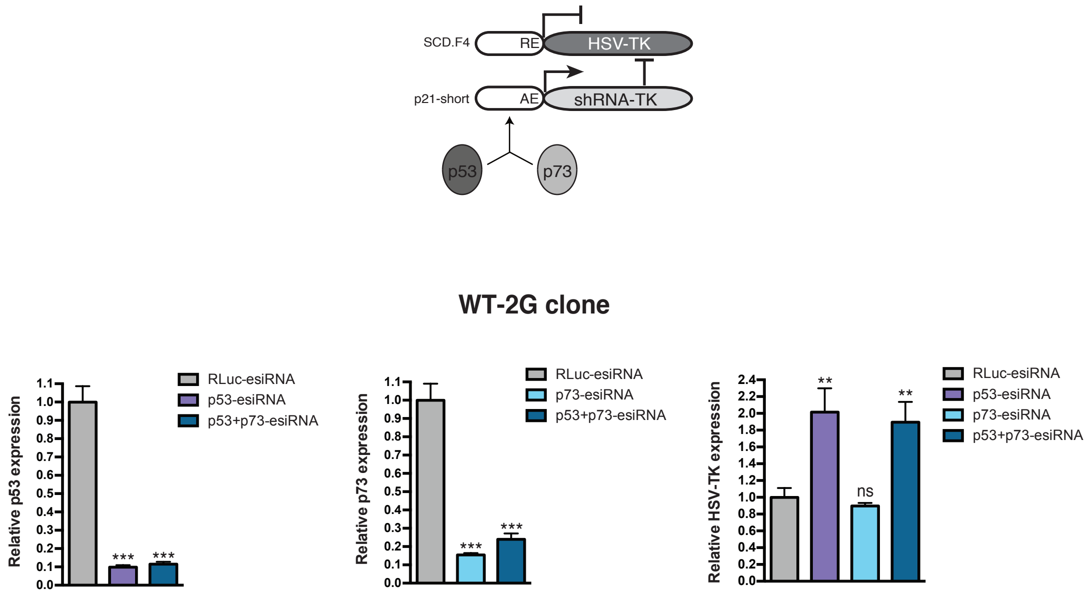

Supplementary figure 2: The 2G sensor is responsive to endogenous levels of p53, but not p73. Top; scheme of the p53 repressive network with HSV-TK as the primary output. Bottom; p53 and p73 were knocked down in WT-2G cells either separately or in tandem using esiRNAs. The left and the middle plot show the extent of p53 and p73 downregulation, respectively, while the right bar plot depicts the increase in HSV-TK expression only upon p53 knock down, but not p73 knock down. A non-targeting (RLuc-esiRNA) silencing trigger served as control. All expression levels were determined by qPCR. Error bars represent SD of 3 independent experiments and Student's two-tailed t-test values are given (*** P<0.001, ** P<0.01 and ns – not significant).

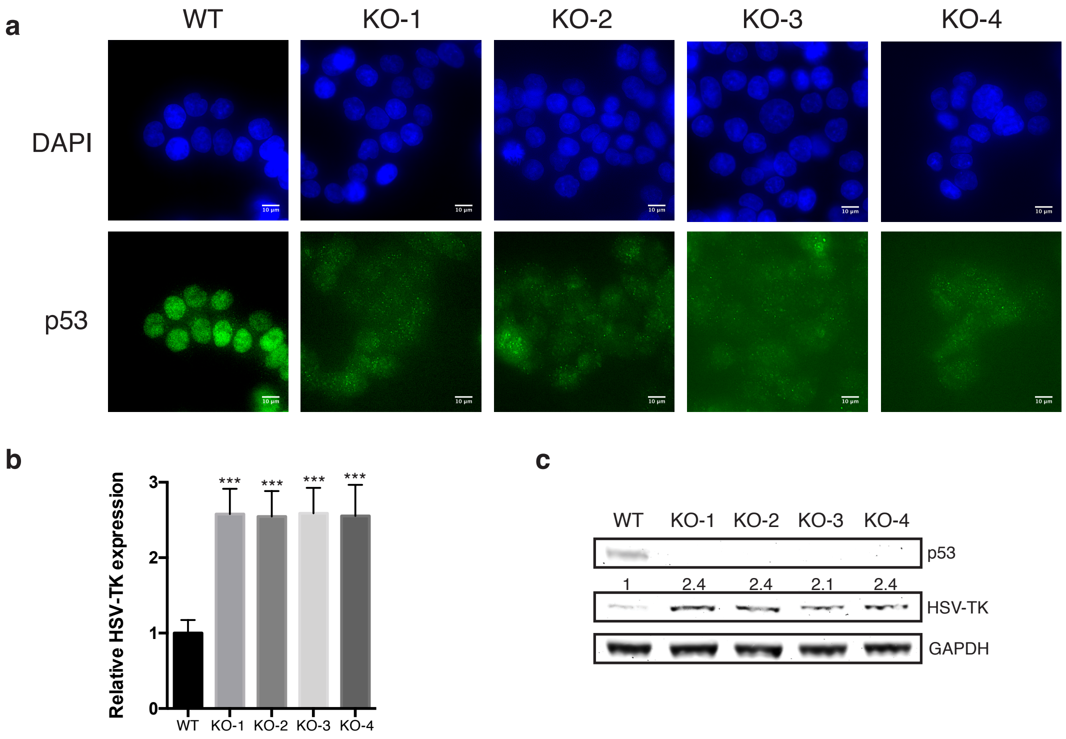

Supplementary figure 3: Different KO clones derived from the WT-2G clone all exhibit increased HSV-TK expression. (**a**) Representative immunofluorescence (IF) images of DAPI (top panel) and p53 antibody-stained (bottom panel) WT-2G cells and four KO clones. Scale bars represent 10 μm. (**b**) Comparison of HSV-TK expression of WT-2G and four KO clones as determined by qPCR. KO-1 clone was used in all subsequent experiments and was termed KO-2G. Error bars depict SD of 3 independent experiments and Student's two-tailed t-test values are given (*** P<0.001). (**c**) Western blot shows upregulation of HSV-TK protein levels in four different KO clones derived from WT-2G clone. The relative quantification of the HSV-TK band signals is provided.

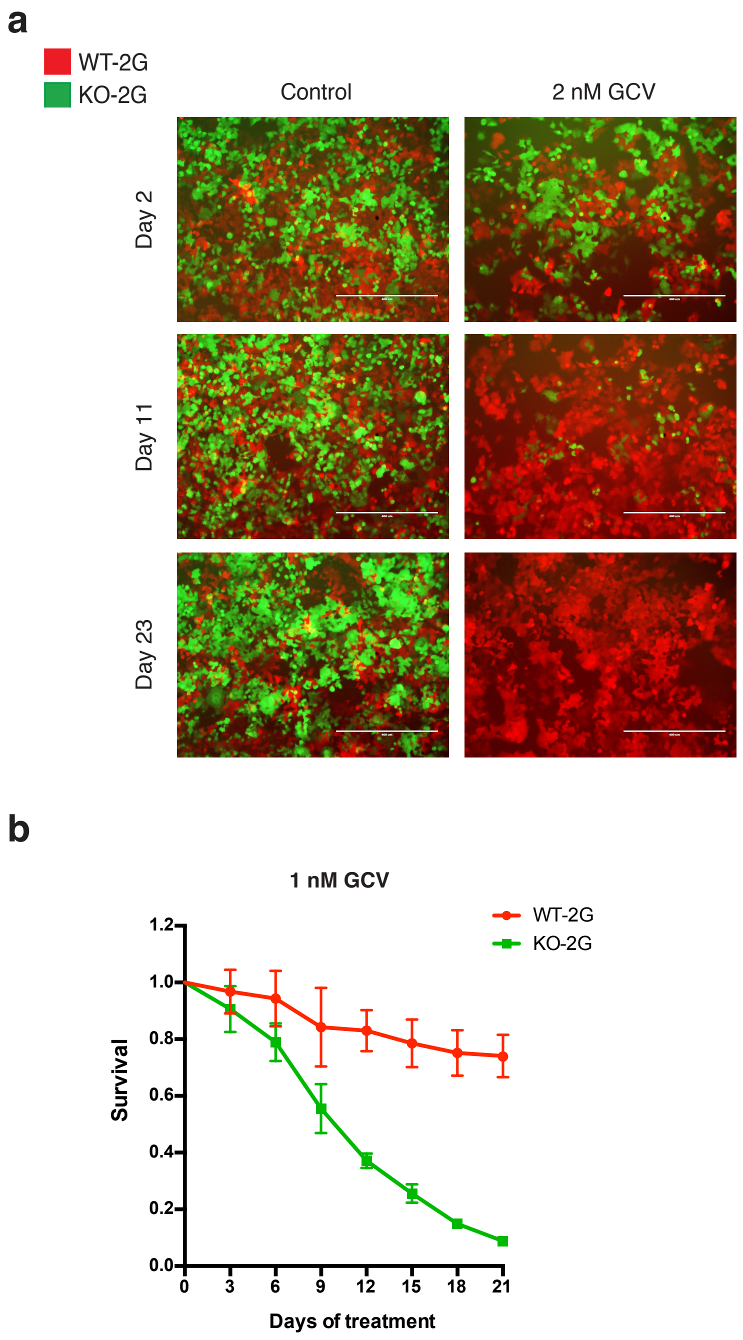

Supplementary figure 4: Low Ganciclovir concentration targets almost exclusively KO-2G cells. (**a**) Representative images of the two-color assay in which a mix of WT-2G (m-Cherry tagged) and KO-2G (GFP-tagged) cells was either treated with 2 nM GCV or control (water) for indicated period of time. Scale bars represent 400 μm. (**b**) WT-2G and KO-2G cells were separately treated with 1 nM Ganciclovir over a period of time. Scatter plot shows the ratio of cell number in treatment versus control group (water) for both cell types during 21 days. Error bars depict SD of 3 independent experiments.

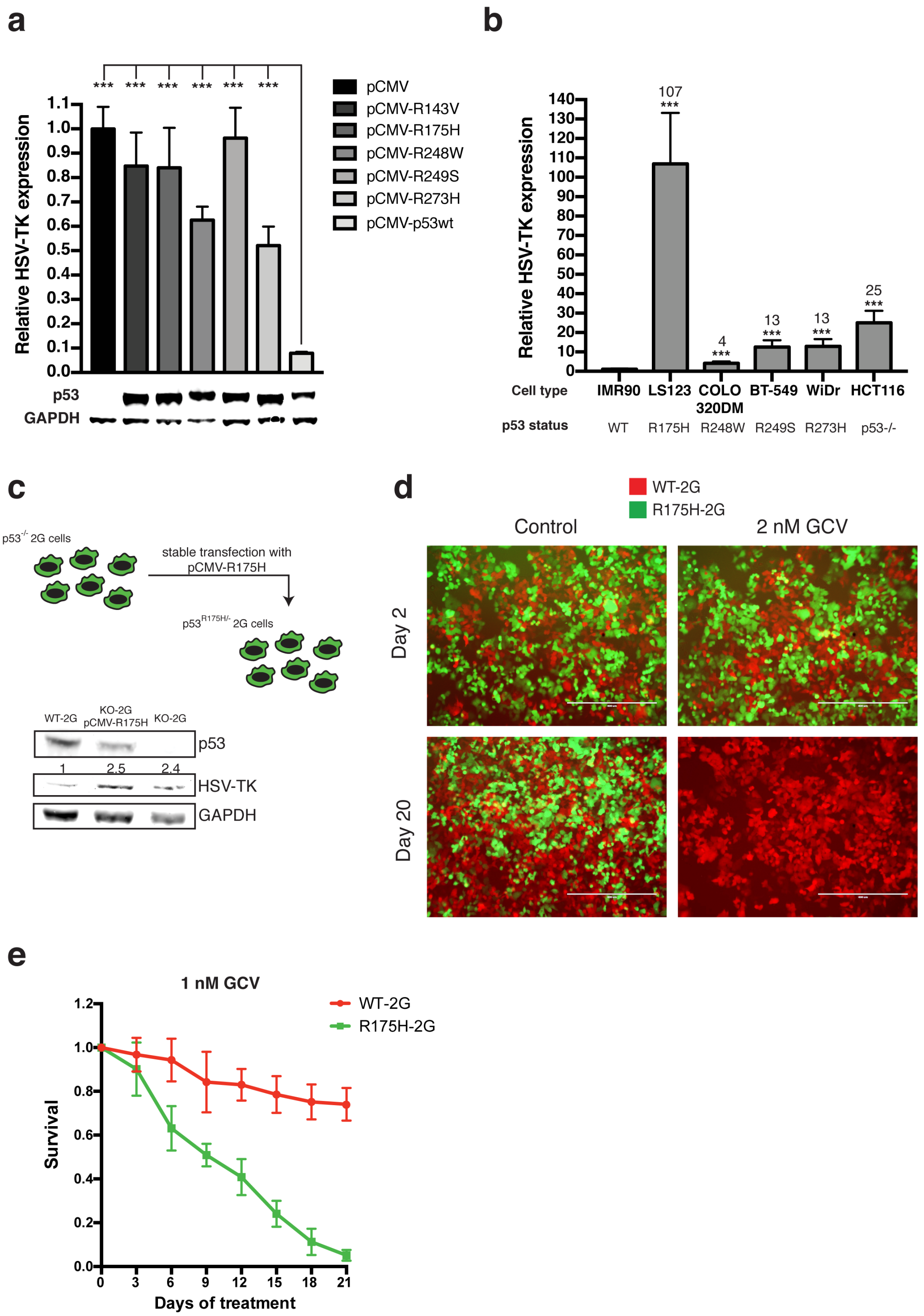
a
b
c
d
e
Relative HSV-TK expression
pCMV
pCMV-R143V
pCMV-R175H
pCMV-R248W
pCMV-R249S
pCMV-R273H
pCMV-p53wt
p53
GAPDH
Cell type
IMR90 LS123 COLO 320DM BT-549 WiDr HCT116
p53 status
WT R175H R248W R249S R273H p53-/-
107
4
13
13
25
p53-/- 2G cells
stable transfection with pCMV-R175H
p53R175H/- 2G cells
WT-2G
KO-2G pCMV-R175H
KO-2G
1
2.5
2.4
HSV-TK
WT-2G
R175H-2G
Control
2 nM GCV
Day 2
Day 20
1 nM GCV
Survival
Days of treatment

Supplementary figure 5: The 2G sensor detects a mutant version of p53 and sensitizes the cells expressing the mutant to Ganciclovir. (**a** and **b**) SCD.F4-HSV-TK and p21-short-shRNA (against HSV-TK) were transiently expressed and HSV-TK expression was measured by qPCR. (**a**) p53 mutants fail to effectively repress the sensor in co-transfection experiments. The bar plot depicts HSV-TK expression of the sensor cotransfected with either WT p53 or five p53 hot spot mutants in HCT116 p53 KO cells. Western blot shows protein levels of WT and mutant p53 and GAPDH as a loading control below the graph. All error bars represent SD of 3 independent experiments and Student's two- tailed t-test values are given (*** $P<0.001$), comparing HSV-TK expression between p53 WT cotransfection and each of the mutants'. (**b**) Cancer cell lines harboring p53 mutations are vulnerable to the sensor. The bar plot depicts HSV-TK expression of the sensor in p53 WT primary fibroblasts (IMR90) and five cell lines with p53 alterations (LS123, COLO320DM, WiDr and HCT116 are colorectal adenocarcinoma cells, while BT-549 are mammary gland ductal carcinoma cells). The ratios of HSV-TK expression compared to the primary fibroblasts are indicated above each bar. The p53 status for each cell line is shown below the graph. All error bars represent SD of 3 independent experiments and Student's two- tailed t-test values are given (*** $P<0.001$), comparing HSV-TK expression between IMR90 (p53 WT) and each of the mutant-expressing cell lines. (**c**) Top; schematic representation of stable integration of p53 R175H overexpression construct in KO-2G clone. Botoom; western blot showing that stably expressed R175H p53 mutant cannot repress HSV-TK levels. The relative quantification of the HSV-TK band signals is provided. (**d**) Representative images of the two-color assay in which a mix of WT-2G (m-Cherry tagged) and KO-2G-R175H (R175H, GFP-tagged) cells was either treated with 2 nM GCV or control (water) for indicated period of time. Scale bars represent 400 μm. (**e**) WT-2G and KO-2G-R175H cells were separately treated with 1 nM Ganciclovir over a period of time. Scatter plot shows the ratio of cell number in treatment versus control group (water) for both cell types during 21 days. Error bars depict SD of 3 independent experiments.

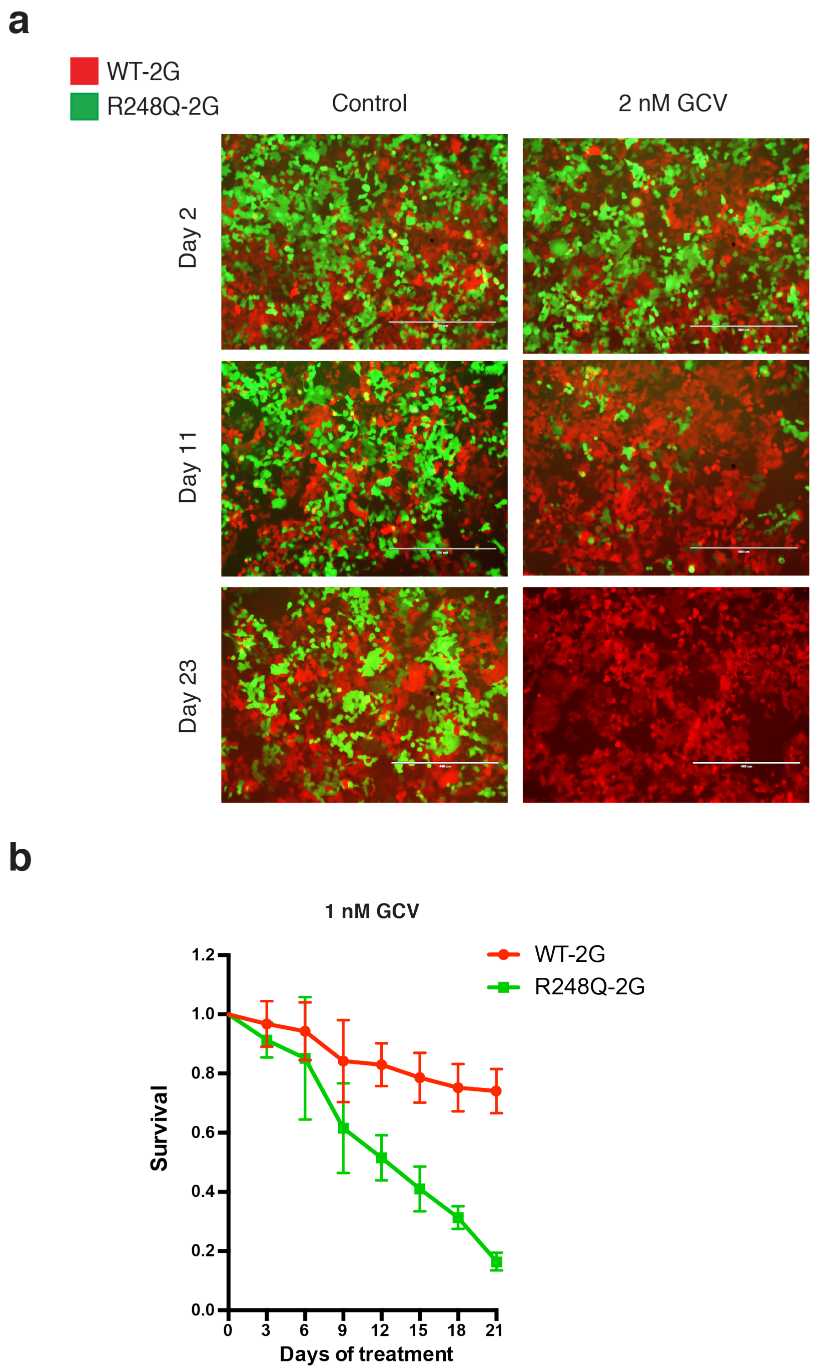

Supplementary figure 6: Low Ganciclovir concentration targets almost exclusively R248Q cells. (**a**) Representative images of the two-color assay in which a mix of WT-2G (m-Cherry tagged) and R248Q-2G (GFP-tagged) cells was either treated with 2 nM GCV or control (water) for indicated period of time. Scale bars represent 400 μm. (**b**) WT-2G and R248Q-2G cells were separately treated with 1 nM Ganciclovir over a period of time. Scatter plot shows the ratio of cell number in treatment versus control group (water) for both cell types during 21 days. Error bars depict SD of 3 independent experiments.

a

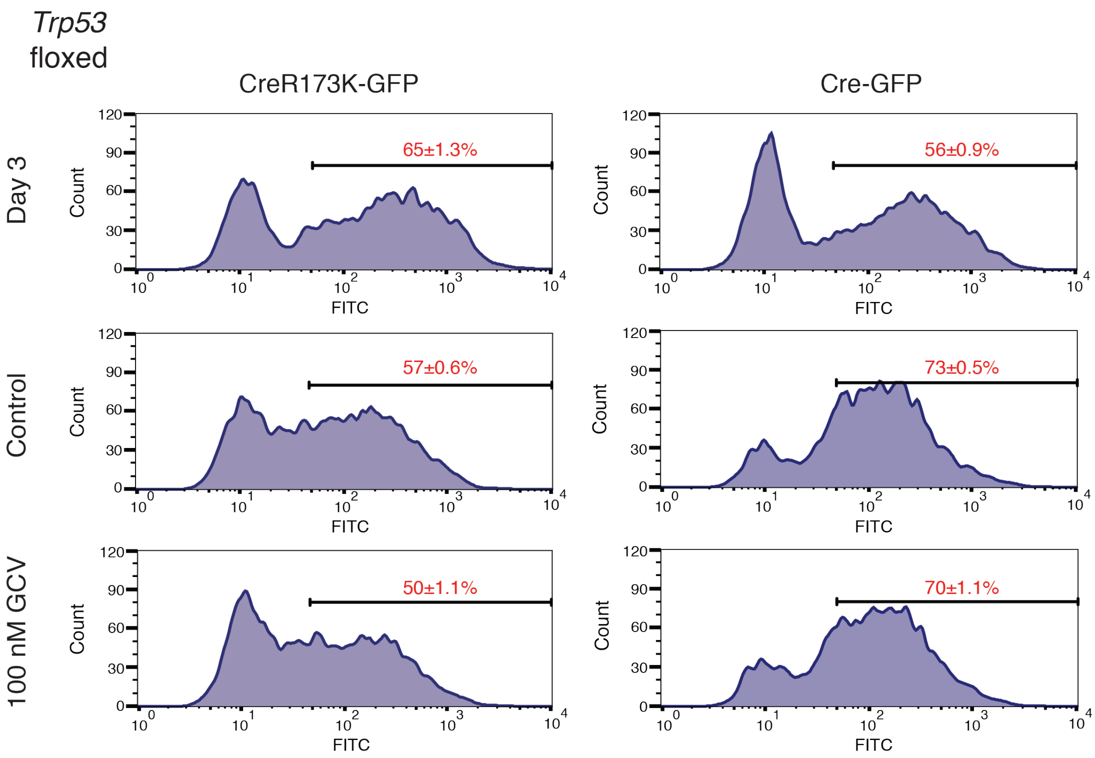
Trp53 floxed
CreR173K-GFP
Cre-GFP
Day 3
Control
100 nM GCV
Count
FITC
65±1.3%
56±0.9%
57±0.6%
73±0.5%
50±1.1%
70±1.1%

b

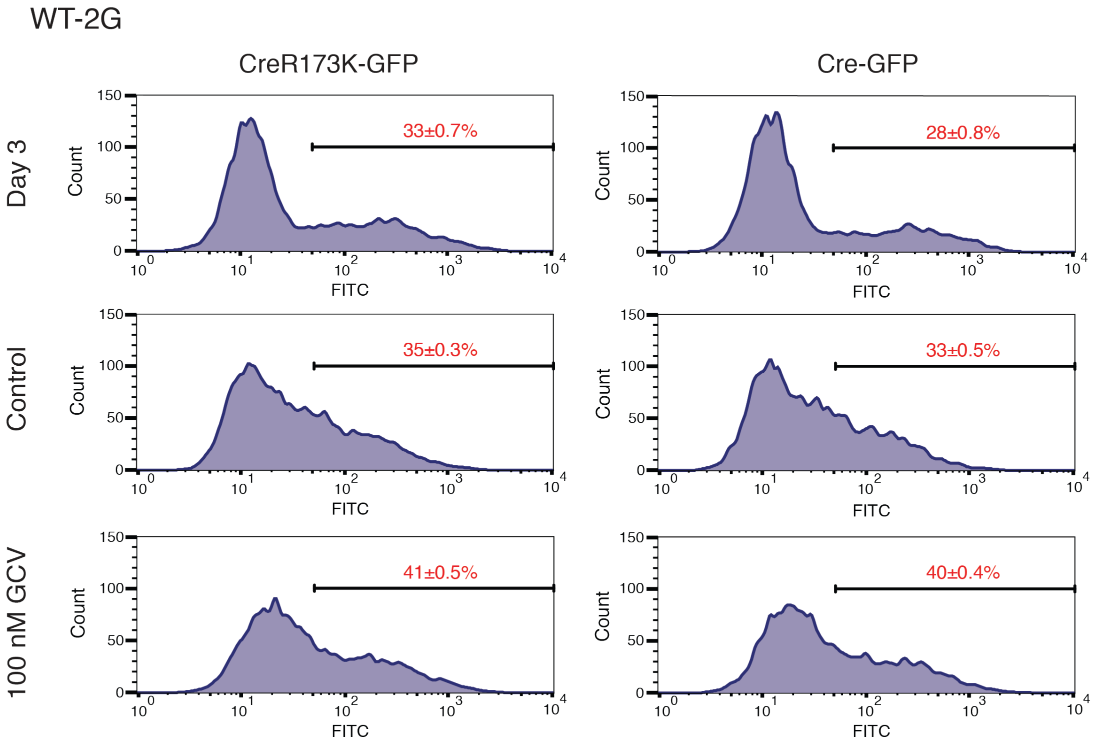
WT-2G
CreR173K-GFP
Cre-GFP
Day 3
Control
100 nM GCV
Count
FITC
33±0.7%
28±0.8%
35±0.3%
33±0.5%
41±0.5%
40±0.4%

Supplementary figure 7: Controls for *Trp53* floxed MEF experiments. FACS histograms depicting the distribution of GFP intensity (GFP positive values are marked with the scale bar) for *Trp53* floxed MEFs (top panel) and WT-2G MEFs (bottom panel) upon transduction with bicistronic Cre-GFP retrovirus (right-hand side) or inactive Cre-GFP retrovirus (left-hand side) in the presence of 5 μM Nutlin-3. The distribution of GFP intensity is shown three days post transduction (top row), and after 6 days of additional control treatment (middle row) or 100 nM GCV treatment (bottom row). Mean percentages of GFP positive cells of three replicates are shown.

a

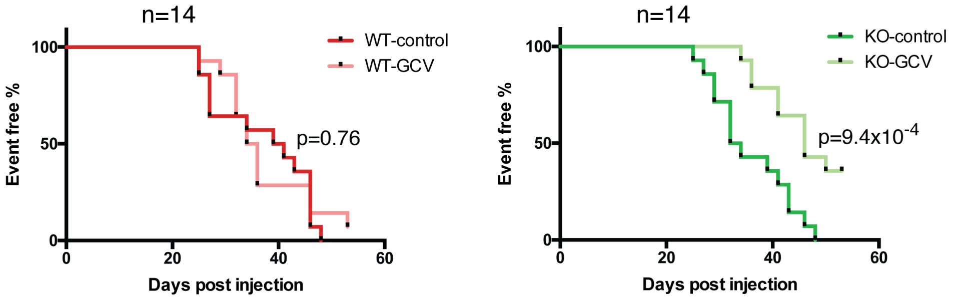

b

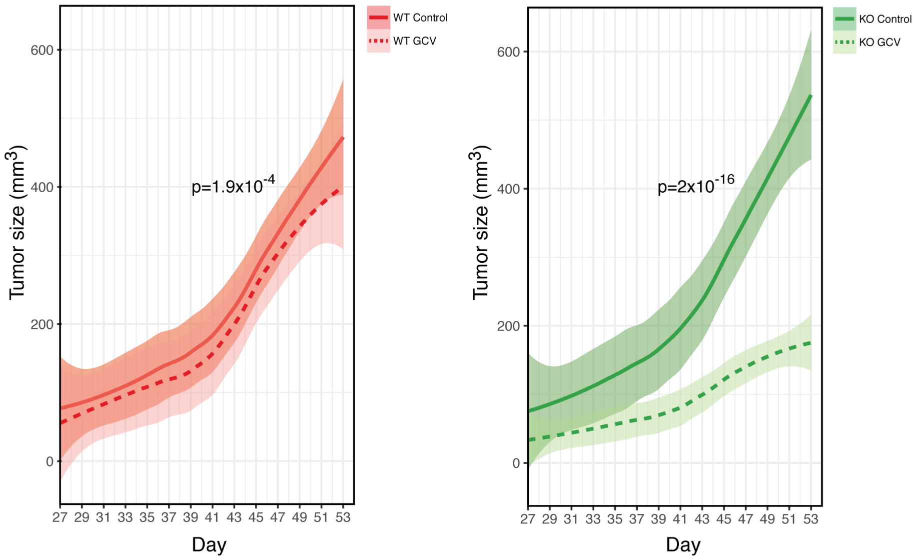

Supplementary figure 8: The sensor specifically targets KO-2G cells *in vivo*. (**a**) Kaplan-Meier graphs show the proportion of mice without palpable tumors (set volume of 100 mm^3) as a function of post-injection time (in days). The left graph compares palpability of WT-2G tumours between mice tretaed with water (n=14) and GCV (n=14), while the right graph compares palpability of KO-2G tumours between the same two groups. P values comparing palpability of WT-2G (control vs. GCV) and KO-2G (control vs. GCV) tumors are given. (**b**) Comparison of average tumour volumes of WT-2G (left) and KO-2G (right) tumours treated with either control (water) or GCV. The curves show results of loess regressions of tumor sizes over time (span = 0.75) and the semi-transparent ribbons indicate 0.95 confidence intervals around the smooth. Indicated p-values are results of likelihood ratio tests comparing linear mixed effects models to respective nested models without an effect of cell type on tumor size.

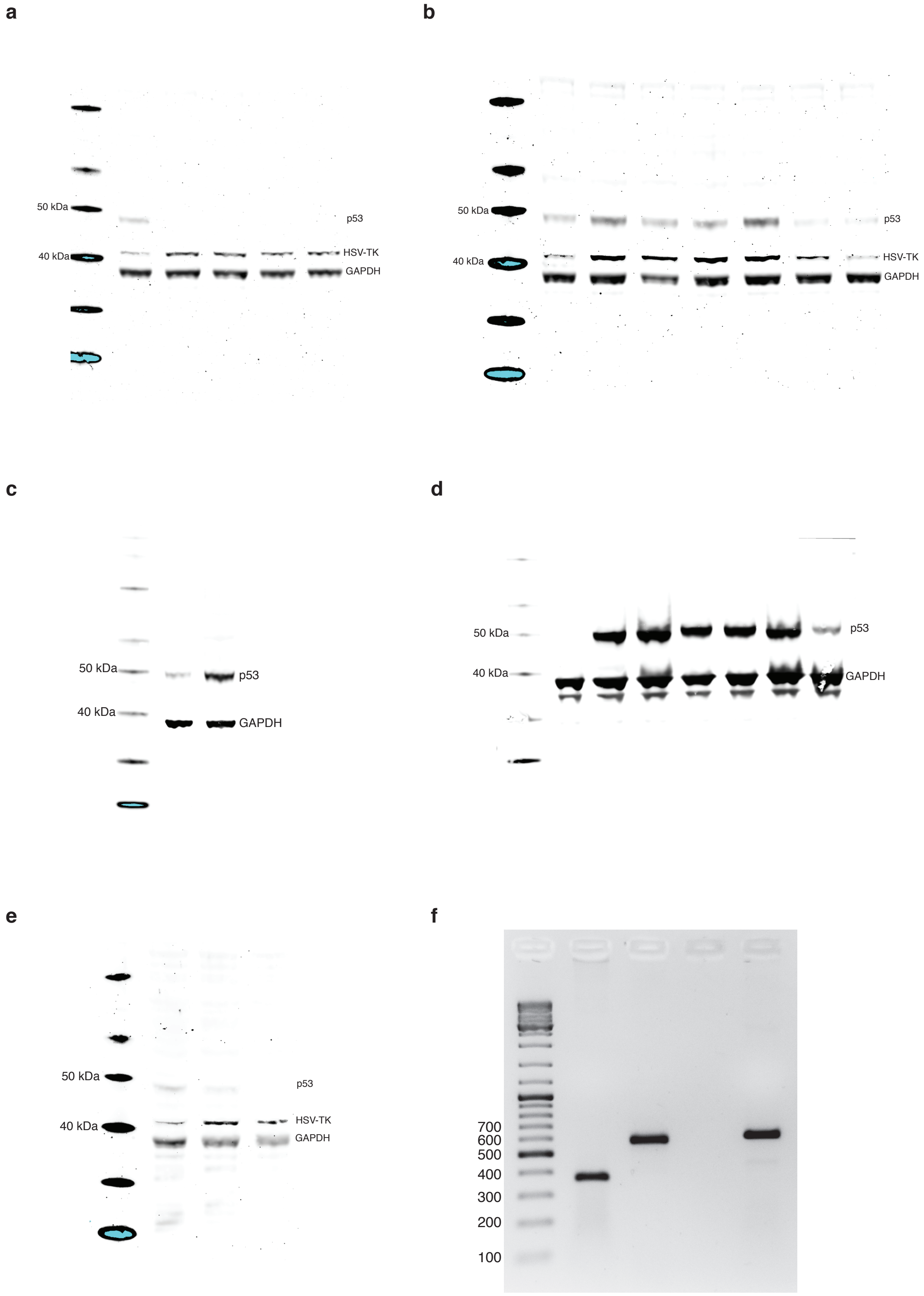
a
50 kDa
40 kDa
p53
HSV-TK
GAPDH
b
50 kDa
40 kDa
p53
HSV-TK
GAPDH
c
50 kDa
40 kDa
p53
GAPDH
d
50 kDa
40 kDa
p53
GAPDH
e
50 kDa
40 kDa
p53
HSV-TK
GAPDH
f
700
600
500
400
300
200
100

Supplementary figure 9: Uncropped versions of Western blots and the agarose gel used in the study. (**a**) Western blot from **Fig. 2c** and **Supporting Fig. 3c**. (**b**) Western blot from **Fig. 3c**. (**c**) Western blot from **Supporting Fig. 1b**. (**d**) Western blot from **Supporting Fig. 5a**. (**e**) Western blot from **Supporting Fig. 5c**. (**f**) Agarose gel electrophoretogram from **Fig. 4a**.

Supplementary Table 1. The complete sequence of the 2G plasmid.

2G
ACTCTTCCTTTTTCAATATTATTGAAGCATTTATCAGGGTTATTGTCTCATGAGCGGATACATATTTGAATGTATT
TAGAAAAATAAACAAATAGGGGTTCCGCGCACATTTCCCCGAAAAGTGCCACCTAAATTGTAAGCGTTAATATTTT
GTTAAAATTCGCGTTAAATTTTTGTTAAATCAGCTCATTTTTTAACCAATAGGCCGAAATCGGCAAAATCCCTTAT
AAATCAAAAGAATAGACCGAGATAGGGTTGAGTGTTGTTCCAGTTTGGAACAAGAGTCCACTATTAAAGAACGTGG
ACTCCAACGTCAAAGGGCGAAAAACCGTCTATCAGGGCGATGGCCCACTACGTGAACCATCACCCTAATCAAGTTT
TTTGGGGTCGAGGTGCCGTAAAGCACTAAATCGGAACCCTAAAGGGAGCCCCCGATTTAGAGCTTGACGGGGAAAG
CCGGCGAACGTGGCGAGAAAGGAAGGGAAGAAAGCGAAAGGAGCGGGCGCTAGGGCGCTGGCAAGTGTAGCGGTCA
CGCTGCGCGTAACCACCACACCCGCCGCGCTTAATGCGCCGCTACAGGGCGCGTCCCATTCGCCATTCAGGCTGCG
CAACTGTTGGGAAGGGCGATCGGTGCGGGCCTCTTCGCTATTACGCCAGCTGGCGAAAGGGGGATGTGCTGCAAGG
CGATTAAGTTGGGTAACGCCAGGGTTTTCCCAGTCACGACGTTGTAAAACGACGGCCAGTGAGCGCGCCTCGTTCA
TTCACGTTTTTGAACCCGTGGAGGACGGGCAGACTCGCGGTGCAAATGTGTTTTACAGCGTGATGGAGCAGATGAA
GATGCTCGACACGCTGCAGAACACGCAGCTAGATTAACCCTAGAAAGATAATCATATTGTGACGTACGTTAAAGAT
AATCATGCGTAAAATTGACGCATGTGTTTTATCGGTCTGTATATCGAGGTTTATTTATTAATTTGAATAGATATTA
AGTTTTATTATATTTACACTTACATACTAATAATAAATTCAACAAACAATTTATTTATGTTTATTTATTTATTAAA
AAAAAACAAAAACTCAAAATTTCTTCTATAAAGTAACAAAACTTTTATGAGGGACAGCCCCCCCCCAAAGCCCCCA
GGGATGTAATTACGTCCCTCCCCCGCTAGGGGGCAGCAGCGAGCCGCCCGGGGCTCCGCTCCGGTCCGGCGCTCCC
CCCGCATCCCCGAGCCGGCAGCGTGCGGGGACAGCCCGGGCACGGGGAAGGTGGCACGGGATCGCTTTCCTCTGAA
CGCTTCTCGCTGCTCTTTGAGCCTGCAGACACCTGGGGGGATACGGGGAAAAGGCCTCCAAGGCCactagtTCTTG
TGAATTGGCTTGCAGTAATAATTCAATACCTGCCAGCTATTCTTATTCCACATCCAAGCCCTTTCGCCTGCTGCTG
GGTGAAAACACATGTCAGTGTTTCCTGACGGTTTCCACAAAGAAGATTCCAAAATTACAACCTGCCAGTCTGAAGA
ATCTCCAAAACATCCCGCACGCATCCTGGAGGCGCGGGCTTGGGGATGGGACTGCCCGCCCGGGTCCTGAACAGGA
TGCGTGCGCGCAGGCACACACACACCAGCCAGCCTGTGTGTGCGGCCGGAGTCCGGTGCGGTCCCGGGTGAGCAGC
GCGTGGCTGGTGGGCGGGGCAGAGCCATTGTTCGCAGGCGTACCGAGTCCCCCGCGCTCGCCCGGGAGGGAGGCGG
GGCTTCCCGCGTCCCCCAAGCTCCAGATCCTGGGGTGGCTGCCACGTCTCCCTGCCACGCGCCTGGGGGGACGGAA
GACGGGACGGAGATGTTAGTGGTGGGCGCCCCCCGAGGGTTCACCACTGTTTCCTGAGAAACTTCCCCAGTGCCCA
CCCACCCGTTCTCCGTGTGCCCGAGGGCCGGTCCTGGGCTAGGCTCCGCGCCCCAGCCCCAAACCGGGTCCCCAGC
CCCTTCCAGAGAGAAAGCTCCCGACGCGGGATGCCGGGCAGAGGCCCAGCGGCGGGTGGAAGAGAAGCTGAGAAGG
AGAAACAGAGGGGAGGGGGAGCGAGGAGCTGGCGGCAGAGGGAACAGCAGATTGCGCCGAGCCAATGGCAACGGCA
GGACGAGGTGGCACCAAATTCCCTTCGGCCAATGACGAGCCGGAGTTTACAGAAGCCTCATTAGCATTTCCCCAGA
GGCAGGGGCAGGGGCAGAGGCCGGGTGGTGTGGTGTCGGTGTCGGCAGCATCCCCGGCGCCCTGCTGCGGTCGCCG
CGAGCCTCGGCCTCTGTCTCCTCCCCCTCCCGCCCTTACCTCCACGCGGGACCGCCCGCGCCAGTCAACTCCTCGC
ACTTTGCCCCTGCTTGGCAGCGGATAAAAGGGGGCTGAGGAAATACCGGACACGGTCACCCGTTGCCAGCTCTAGC
CTTTAAATTCCCGGCTCGGGGACCTCCACGCACCGCGGCTAGCGCCGACAACCAGCTAGCGTGCAAGGCGCCGCGG
CTCAGCGCGTACCGGCGGGCTTCGAAACCGCAGTCCTCCGGCGACCCCGAACTCCGCTCCGGAGCCTCAGCCCCCT
GGAAAGTGATCCCGGCATCCGAGAGCCAAGATCCCGGCCCACTTGCTGCAGGACGTTGTGAGTTTCCCAGCCTGGC
CCCGTACCGCCGGGTCGCAGGCGCGGGCTGGGCTTCCAGGGGACGGGTTGGTGGCAGAAGAGAGGGGAGAGCTCCG
CGGAGGACTTGGTCATCTTTTTCGAGTTGTGCTGCCTTCCGTGAGTTGGGAAAGTGGATTGTAATTTGGGGACTTG
AGTCTCCAACTTTAGTTTCTTAAGCTTTAAAGAAAAATCCGGTCGTGCTGGTGCTTTTAAGAATTAAGCGGGTTTT
CCTTTGTCTTCGTGGGGAAGTGAGTGCTTTACTTCTCCTTCCTACTGCCGCCTCCCGATAGGTTTCGCGCCCCTCG
TCCCCCTCGCCCTCCGCCCCTATTGTATCTGTACAGTTTCAGGGAACTTTTCTCCGTTGCGTCTCGGATACACCCT
ACCCTCAGTGAACTACGGCGCTGCGGAAGGGTCCGTACTGTCCACCCTTCCCCCAGCGTGATTAGAGAGCGGAGTG
GCCCCAGCTGCCTCCACGTGTCTCTTCTCCTGACTCTCCTCTTCTCGAGATCTGCGATCTAAGTAAGCTTATGGCT
TCGTACCCCTGCCATCAACACGCGTCTGCGTTCGACCAGGCTGCGCGTTCTCGCGGCCATAGCAACCGACGTACGG
CGTTGCGCCCTCGCCGGCAGCAAGAAGCCACGGAAGTCCGCCTGGAGCAGAAAATGCCCACGCTACTGCGGGTTTA
TATAGACGGTCCTCACGGGATGGGGAAAACCACCACCACGCAACTGCTGGTGGCCCTGGGTTCGCGCGACGATATC
GTCTACGTACCCGAGCCGATGACTTACTGGCAGGTGCTGGGGGCTTCCGAGACAATCGCGAACATCTACACCACAC
AACACCGCCTCGACCAGGGTGAGATATCGGCCGGGGACGCGGCGGTGGTAATGACAAGCGCCCAGATAACAATGGG
CATGCCTTATGCCGTGACCGACGCCGTTCTGGCTCCTCATATCGGGGGGGAGGCTGGGAGCTCACATGCCCCGCCC
CCGGCCCTCACCCTCATCTTCGACCGCCATCCCATCGCCGCCCTCCTGTGCTACCCGGCCGCGCGATACCTTATGG
GCAGCATGACCCCCCAGGCCGTGCTGGCGTTCGTGGCCCTCATCCCGCCGACCTTGCCCGGCACAAACATCGTGTT
GGGGGCCCTTCCGGAGGACAGACACATCGACCGCCTGGCCAAACGCCAGCGCCCCGGCGAGCGGCTTGACCTGGCT
ATGCTGGCCGCGATTCGCCGCGTTTACGGGCTGCTTGCCAATACGGTGCGGTATCTGCAGGGCGGCGGGTCGTGGC
GGGAGGATTGGGGACAGCTTTCGGGGACGGCCGTGCCGCCCCAGGGTGCCGAGCCCCAGAGCAACGCGGGCCCACG
ACCCCATATCGGGGACACGTTATTTACCCTGTTTCGGGCCCCCGAGTTGCTGGCCCCCAACGGCGACCTGTACAAC
GTGTTTGCCTGGGCCTTGGACGTCTTGGCCAAACGCCTCCGTCCCATGCACGTCTTTATCCTGGATTACGACCAAT
CGCCCGCCGGCTGCCGGGACGCCCTGCTGCAACTTACCTCCGGGATGGTCCAGACCCACGTCACCACCCCCGGCTC
CATACCGACGATCTGCGACCTGGCGCGCACGTTTGCCCGGGAGATGGGGGAGGCTAACTGAGTCTAGACACCTACG
GCAAGCTGACCCTGAAGTTCATCTGCACCAGCAAGCTGACCCTGAAGTTCATCACCTACGGCAAGCTGACCCTGAA
GTTCATCTGCACCAGGCCGGCCGCTTCGAGCAGACATGATAAGATACATTGATGAGTTTGGACAAACCACAACTAG
AATGCAGTGAAAAAAATGCTTTATTTGTGAAATTTGTGATGCTATTGCTTTATTTGTAACCATTATAAGCTGCAAT
AAACAAGTTAACAACAACAATTGCATTCATTTTATGTTTCAGGTTCAGGGGGAGGTGTGGGAGGTTTTTTAAAGCA
AGTAAAACCTCTACAAATGTGGTAAAATCGgaattcgaatttaaatcggatccgcggccgcgaaggatctgcgatc
gctccggtgcccgtcagtgggcagagcgcacatcgcccacagtccccgagaagttggggggaggggtcggcaattg
aacgggtgcctagagaaggtggcgcggggtaaactgggaaagtgatgtcgtgtactggctccgcctttttcccgag

ggtgggggagaaccgtatataagtgcagtagtcgccgtgaacgttctttttcgcaacgggtttgccgccagaacac
agctgaagcttcgaggggctcgcatctctccttcacgcgcccgccgccctacctgaggccgccatccacgccggtt
gagtcgcgttctgccgcctcccgcctgtggtgcctcctgaactgcgtccgccgtctaggtaagtttaaagctcagg
tcgagaccgggcctttgtccggcgctcccttggagcctacctagactcagccggctctccacgctttgcctgaccc
tgcttgctcaactctacgtctttgtttcgttttctgttctgcgccgttacagatccaagctgtgaccggcgcctac
gctagatgaccgagtacaagcccacggtgcgcctcgccacccgcgacgacgtccccagggccgtacgcaccctcgc
cgccgcgttcgccgactaccccgccacgcgccacaccgtcgatccggaccgccacatcgagcgggtcaccgagctg
caagaactcttcctcacgcgcgtcgggctcgacatcggcaaggtgtgggtcgcggacgacggcgccgcggtggcgg
tctggaccacgccggagagcgtcgaagcgggggcggtgttcgccgagatcggcccgcgcatggccgagttgagcgg
ttcccggctggccgcgcagcaacagatggaaggcctcctggcgccgcaccggcccaaggagcccgcgtggttcctg
gccaccgtcggcgtctcgcccgaccaccagggcaagggtctgggcagcgccgtcgtgctccccggagtggaggcgg
ccgagcgcgccggggtgcccgccttcctggagacctccgcgccccgcaacctccccttctacgagcggctcggctt
caccgtcaccgccgacgtcgaggtgcccgaaggaccgcgcacctggtgcatgacccgcaagcccggtgcctgagtc
gacaatcaacctctggattacaaaatttgtgaaagattgactggtattcttaactatgttgctccttttacgctat
gtggatacgctgctttaatgcctttgtatcatGTTAACTTGTTTATTGCAGCTTATAATGGTTACAAATAAAGCAA
TAGCATCACAAATTTCACAAATAAAGCATTTTTTTCACTGCATTCTAGTTGTGGTTTGTCCAAACTCATCAATGTA
TCTTATCATGTCTGGAATTGACTCAAATGATGTCAATTAGTCTATCAGAAGCTATCTGGTCTCCCTTCCGGGGGAC
AAGACATCCCTGTTTAATATTTAAACAGCAGTGTTCCCAAACTGGGTTCTTATATCCCTTGCTCTGGTCAACCAGG
TTGCAGGGTTTCCTGTCCTCACAGGAACGAAGTCCCTAAAGAAACAGTGGCAGCCAGGTTTAGCCCCGGAATTGAC
TGGATTCCTTTTTTAGGGCCCATTGGTATGGCTTTTTCCCCGTATCCCCCCAGGTGTCTGCAGGCTCAAAGAGCAG
CGAGAAGCGTTCAGAGGAAAGCGATCCCGTGCCACCTTCCCCGTGCCCGGGCTGTCCCCGCACGCTGCCGGCTCGG
GGATGCGGGGGGAGCGCCGGACCGGAGCGGAGCCCCGGGCGGCTCGCTGCTGCCCCCTAGCGGGGGAGGGACGTAA
TTACATCCCTGGGGGCTTTGGGGGGGGGCTGTCCCTGATATCTATAACAAGAAAATATATATATAATAAGTTATCA
CGTAAGTAGAACATGAAATAACAATATAATTATCGTATGAGTTAAATCTTAAAAGTCACGTAAAAGATAATCATGC
GTCATTTTGACTCACGCGGTCGTTATAGTTCAAAATCAGTGACACTTACCGCATTGACAAGCACGCCTCACGGGAG
CTCCAAGCGGCGACTGAGATGTCCTAAATGCACAGCGACGGATTCGCGCTATTTAGAAAGAGAGAGCAATATTTCA
AGAATGCATAGGGACAGCCCCCCCCCAAAGCCCCCAGGGATGTAATTACGTCCCTCCCCCGCTAGGGGGCAGCAGC
GAGCCGCCCGGGGCTCCGCTCCGGTCCGGCGCTCCCCCCGCATCCCCGAGCCGGCAGCGTGCGGGGACAGCCCGGG
CACGGGGAAGGTGGCACGGGATCGCTTTCCTCTGAACGCTTCTCGCTGCTCTTTGAGCCTGCAGACACCTGGGGGG
ATACGGGGAAAAGGCCTCCACGGCCactagtAGCAGGCTGTGGCTCTGATTGGCTTTCTGGCCATTAGGAACATGT
CCCAACATGTTGAGCTCTGGCATAGAAGAGGCTGGTGGCTATTTTGTCCTTGGCTAGCCCGGGCTCGAGATCTGCG
ATCTAAGTAAGCTTGAATATTGCTGTTTGAATGAGGCTTCAGTACTTTACAGAATCGTTGCCTGCACATCTTGGAA
ACACTTGCTGGGATTACTTCTTCAGGTTAACCCAACAGAAGGCTAAAGAAGGTATATTGCTGTTGACAGTGAGCGG
CAAGCTGACCCTGAAGTTCATTAGTGAAGCCACAGATGTAATGAACTTCAGGGTCAGCTTGTTGCCTACTGCCTCG
GACTTCAAGGGGCTACTTTAGGAGCAATTATCTTGTTTACTAAAACTGAATACCTTGCTATCTCTTTGATACATTT
TTACAAAGCTGAATTAAAATGGTATAAATTAAATCACTTTAAAACCATGTCTGTCTAGAGTCGGGGCGGCCGGCCG
CTTCGAGCAGACATGATAAGATACATTGATGAGTTTGGACAAACCACAACTAGAATGCAGTGAAAAAAATGCTTTA
TTTGTGAAATTTGTGATGCTATTGCTTTATTTGTAACCATTATAAGCTGCAATAAACAAGTTAACAACAACAATTG
CATTCATTTTATGTTTCAGGTTCAGGGGGAGGTGTGGGAGGTTTTTTAAAGCAAGTAAAACCTCTACAAATGTGGT
AAAATCGgaattcTCCTCACAGGAACGAAGTCCCTAAAGAAACAGTGGCAGCCAGGTTTAGCCCCGGAATTGACTG
GATTCCTTTTTTAGGGCCCATTGGTATGGCTTTTTCCCCGTATCCCCCCAGGTGTCTGCAGGCTCAAAGAGCAGCG
AGAAGCGTTCAGAGGAAAGCGATCCCGTGCCACCTTCCCCGTGCCCGGGCTGTCCCCGCACGCTGCCGGCTCGGGG
ATGCGGGGGGAGCGCCGGACCGGAGCGGAGCCCCGGGCGGCTCGCTGCTGCCCCCTAGCGGGGGAGGGACGTAATT
ACATCCCTGGGGGCTTTGGGGGGGGGCTGTCCCTATGCATGCGTCAATTTTACGCAGACTATCTTTCTAGGGTTAA
TCTAGCTGCATCAGGATCATATCGTCGGGTCTTTTTTCCGGCTCAGTCATCGCCCAAGCTGGCGCTATCTGGGCAT
CGGGGAGGAAGAAGCCCGTGCCTTTTCCCGCGAGGTTGAAGCGGCATGGAAAGAGTTTGCCGAGGATGACTGCTGC
TGCATTGACGTTGAGCGAAAACGCACGTTTACCATGATGATTCGGGAAGGTGTGGCCATGCACGCCTTTAACGGTG
AACTGTTCGTTCAGGCCACCTGGGATACCAGTTCGTCGCGGCTTTTCCGGACACAGTTCCGGATGGTCAGCCCGAA
GCGCATCAGCAACCCGAACAATACCGGCGACAGCCGGAACTGCCGTGCCGGTGTGCAGATTAATGACAGCGGTGCG
GCGCTGGGATATTACGTCAGCGAGGACGGGTATCCTGGCTGGATGCCGCAGAAATGGACATGGATACCCCGTGAGT
TACCCGGCGGGCGCGCTTGGCGTAATCATGGTCATAGCTGTTTCCTGTGTGAAATTGTTATCCGCTCACAATTCCA
CACAACATACGAGCCGGAAGCATAAAGTGTAAAGCCTGGGGTGCCTAATGAGTGAGCTAACTCACATTAATTGCGT
TGCGCTCACTGCCCGCTTTCCAGTCGGGAAACCTGTCGTGCCAGCTGCATTAATGAATCGGCCAACGCGCGGGGAG
AGGCGGTTTGCGTATTGGGCGCTCTTCCGCTTCCTCGCTCACTGACTCGCTGCGCTCGGTCGTTCGGCTGCGGCGA
GCGGTATCAGCTCACTCAAAGGCGGTAATACGGTTATCCACAGAATCAGGGGATAACGCAGGAAAGAACATGTGAG
CAAAAGGCCAGCAAAAGGCCAGGAACCGTAAAAAGGCCGCGTTGCTGGCGTTTTTCCATAGGCTCCGCCCCCCTGA
CGAGCATCACAAAAATCGACGCTCAAGTCAGAGGTGGCGAAACCCGACAGGACTATAAAGATACCAGGCGTTTCCC
CCTGGAAGCTCCCTCGTGCGCTCTCCTGTTCCGACCCTGCCGCTTACCGGATACCTGTCCGCCTTTCTCCCTTCGG
GAAGCGTGGCGCTTTCTCATAGCTCACGCTGTAGGTATCTCAGTTCGGTGTAGGTCGTTCGCTCCAAGCTGGGCTG
TGTGCACGAACCCCCCGTTCAGCCCGACCGCTGCGCCTTATCCGGTAACTATCGTCTTGAGTCCAACCCGGTAAGA
CACGACTTATCGCCACTGGCAGCAGCCACTGGTAACAGGATTAGCAGAGCGAGGTATGTAGGCGGTGCTACAGAGT
TCTTGAAGTGGTGGCCTAACTACGGCTACACTAGAAGGACAGTATTTGGTATCTGCGCTCTGCTGAAGCCAGTTAC
CTTCGGAAAAAGAGTTGGTAGCTCTTGATCCGGCAAACAAACCACCGCTGGTAGCGGTGGTTTTTTTGTTTGCAAG
CAGCAGATTACGCGCAGAAAAAAAGGATCTCAAGAAGATCCTTTGATCTTTTCTACGGGGTCTGACGCTCAGTGGA
ACGAAAACTCACGTTAAGGGATTTTGGTCATGAGATTATCAAAAAGGATCTTCACCTAGATCCTTTTAAATTAAAA
ATGAAGTTTTAAATCAATCTAAAGTATATATGAGTAAACTTGGTCTGACAGTTACCAATGCTTAATCAGTGAGGCA
CCTATCTCAGCGATCTGTCTATTTCGTTCATCCATAGTTGCCTGACTCCCCGTCGTGTAGATAACTACGATACGGG
AGGGCTTACCATCTGGCCCCAGTGCTGCAATGATACCGCGAGACCCACGCTCACCGGCTCCAGATTTATCAGCAAT
AAACCAGCCAGCCGGAAGGGCCGAGCGCAGAAGTGGTCCTGCAACTTTATCCGCCTCCATCCAGTCTATTAATTGT

```
TGCCGGGAAGCTAGAGTAAGTAGTTCGCCAGTTAATAGTTTGCGCAACGTTGTTGCCATTGCTACAGGCATCGTGG
TGTCACGCTCGTCGTTTGGTATGGCTTCATTCAGCTCCGGTTCCCAACGATCAAGGCGAGTTACATGATCCCCCAT
GTTGTGCAAAAAAGCGGTTAGCTCCTTCGGTCCTCCGATCGTTGTCAGAAGTAAGTTGGCCGCAGTGTTATCACTC
ATGGTTATGGCAGCACTGCATAATTCTCTTACTGTCATGCCATCCGTAAGATGCTTTTCTGTGACTGGTGAGTACT
CAACCAAGTCATTCTGAGAATAGTGTATGCGGCGACCGAGTTGCTCTTGCCCGGCGTCAATACGGGATAATACCGC
GCCACATAGCAGAACTTTAAAAGTGCTCATCATTGGAAAACGTTCTTCGGGGCGAAAACTCTCAAGGATCTTACCG
CTGTTGAGATCCAGTTCGATGTAACCCACTCGTGCACCCAACTGATCTTCAGCATCTTTTACTTTCACCAGCGTTT
CTGGGTGAGCAAAAACAGGAAGGCAAAATGCCGCAAAAAAGGGAATAAGGGCGACACGGAAATGTTGAATACTCAT
```

Supplementary Table 2. Cas9-induced indels in *TP53* locus in KO-2G and heterozygous clone. Newly introduced stop codons are underlined (Ht – heterozygous).

	**Exon 2**	**Exon 4**	**Exon 6**
**WT allele**	TTTCAGACCTATGGAAACTGTGAGTG	TGCTGTCCCCGGA-CGATATTGAACAA	GTGGTGCCCTATGAGCCGCCTGAGGTCTGGT
**KO-2G allele 1**	TTTCAGACCTATG-AAACTGTGAGTG	TGCTGTCCCCGGAACGATATTGAACAA	GTGGTGCCCTATGAGCCGCCTGAGGTCTGGT
**KO-2G allele 2**	TTTCAGACCTATGGAAACTGTGAGTG	TGCTGTCCCCGGA-CGATATTGAACAA	GTGGTGCCC-----------TGAGGTCTGGT
**Ht allele 1**	TTTCAGACCTATGGAAACTGTGAGTG	TGCTGTCCCCGGAACGATATTGAACAA	GTGGTGCCCTATGAGCCGCCTGAGGTCTGGT

Supplementary Table 3. Sequences of primers used to construct luciferase- and HSV-TK-based 2G sensor.

Name of the primers	Forward primer (5’-3’)	Reverse primer (5’-3’)
**CDC25C**	TTTGGTACCGGTCCTCTGGATTGCGATAA	AAAGCTAGCGGACCCTAAGGGGGACAATG
**SCD**	TTTGGTACCTCTTGTGAATTGGCTTGCAG	AAAGCTAGCAGCCGGGAATTTAAAGGCTA
**RGS13**	TTTGGTACCATCTCATTGGGCCCCTAAAT	AAAGCTAGCTTTCCTCTGTTGCCCAACTT
**F1**	ATTTGGTACCGGCAGAGCCATTGTTCGC	TAAACTCGAGCGTCGGGAGCTTTCTCTCTG
**F2**	TTAAGGTACCCCAGAGAGAAAGCTCCCGAC	TTAACTCGAGTATTTCCTCAGCCCCCTTTT
**F3**	ATTTGGTACCCGAGCCGGAGTTTACAGAAG	TAAACTCGAGAAGAGGAGAGTCAGGA
**F4**	ATTTGGTACCTCTTGTGAATTGGCTTGCAG	TAAACTCGAGAAGAGGAGAGTCAGGA
**p21**	TTTGGTACCTCTTGGGAGCCTGTGTGAAG	AAAGCTAGCACAGGCACCTTCTCCCACT
**p21-short**	TTTGGTACCGGCAGCAGGCTGTGGCTCTG	AAAGCTAGCCAAGGACAAAATAGCCACCA
**BS2-PUMA**	TTTGGTACCCGCTGCAGGGAAACCCCCGG	AAAGCTAGCCGCCCCCGCGTGACGCTAC
**4xBS2-PUMA**	TTTGGTACCGAGCTCTTACGCGTGCTAGGCT GCAAGTCCTGACTTGTCCACACTCTGCAAGT CCTGACTTGTCCCTAGGCTGCAAGTCCTGAC TTGTCCACACTCTGCAAGTCCTGACTTGTCC CTAGCCCGGGCTCGACAGCTGGACGTCGATA TCGAATTCGGGTATATAATGGATCCGGTATC GAGATCTGCGATCTAAGTAAGCTTAAA	AAAGCTAGCACTTAGATCGCAGATCTCGATA CCGGATCCATTATATACCCgaattcGATATC GACGTCCAGCTGTCGAGCCCGGGCTAGGGAC AAGTCAGGACTTGCAGAGTGTGGACAAGTCA GGACTTGCAGCCTAGGGACAAGTCAGGACTT GCAGAGTGTGGACAAGTCAGGACTTGCAGCC TAGCACGCGTAAGAGCTCGGTACCAAA
**miR30a**	TTTAAGCTTGAATATTGCTGTTTGAATGAGG	AAATCTAGACAGACATGGTTTTAAAGTGATT TA
**shRNA-FfLuc**	GACGATATGGGCTGAATACAAATAGTGAAGC CACAGATGTATTTGTATTCAGCCCATATCGT TTGCCTACTGCCTCGGACTTC	TGTATTCAGCCCATATCGTCCGCTCACTGTC AACAGCAAT
**shRNA-GFP**	GCAAGCTGACCCTGAAGTTCATTAGTGAAGC CACAGATGTAATGAACTTCAGGGTCAGCTTG TTGCCTACTGCCTCGGACTTC	GAACTTCAGGGTCAGCTTGCCGCTCACTGTC AACAGCAAT
**HSV-TK**	TTTAAGCTTATGGCTTCGTACCCCTGCCA	AAATCTAGACTCAGTTAGCCTCCCCCATCT
**GFP.seed**	TTTTCTAGACACCTACGGCAAGCTGACCCTG AAGTTCATCTGCACCAGCAAGCTGACCCTGA AGTTCATCACCTACGGCAAGCTGACCCTGAA GTTCATCTGCACCAGGCCGGCCTTT	AAAGGCCGGCCTGGTGCAGATGAACTTCAGG GTCAGCTTGCCGTAGGTGATGAACTTCAGGG TCAGCTTGCTGGTGCAGATGAACTTCAGGGT CAGCTTGCCGTAGGTGTCTAGAAAA

Supplementary Table 4. Sequences of gRNAs used in the study.

gRNA	Sequence (5'-3')
TP53 gRNA1	GATCCACTCACAGTTTCCAT
TP53 gRNA2	CCATTGTTCAATATCGTCCG
TP53 gRNA3	GGTGCCCTATGAGCCGCCTG
R248Q gRNA	CCGGTTCATGCCGCCCATGC

Supplementary Table 5. Sequences of primers used to confirm mutations/indels in *TP53* and *Trp53* locus.

Site of induced mutation/indel	Forward primer (5'-3')	Reverse primer (5'-3')
gRNA1/2	CAGCCATTCTTTTCCTGCTC	GGAAGGGACAGAAGATGACA
gRNA3	GCGCTGCTCAGATAGCGAT	GGCCCTTAGCCTCTGTAAGC
R248Q	GGAGAATGGCGTGAACCTGG	GTCAGAGGCAAGCAGAGGCT
Trp53 deletion confirmation primers (MEFs)	**1**: CACAAAAACAGGTTAAACCCAG<br>**3**: AAGGGGTATGAGGGACAAGG	**2**: AGCACATAGGAGGCAGAGAC<br>**4**: GAAGACAGAAAAGGGGAGGG

Supplementary Table 6. Summary of oligonucleotides used for qPCR. In addition, annealing temperatures used to run a qPCR reaction are also listed.

Gene name	Forward primer (5'-3')	Reverse primer (5'-3')	Annealing temperature (°C)
TP53	CCCAAGCAATGGATGATTTGA	GGCATTCTGGGAGCTTCATCT	60
HSV-TK	TGACTTACTGGCAGGTGCTG	GTTATCTGGGCGCTTGTCAT	60
GAPDH	CAGCCTCAAGATCATCAGCA	TGTGGTCATGAGTCCTTCCA	60
TBP	AGGTTAGAAGGCCTTGTGCTC	GGAGAACAATTCTGGGTTTGATCA	60
TP73	CGTGGAAGGCAATAATCTCTC	GTTCATGCCCCCTACACA	60
GFP	GACGTAAACGGCCACAAGTT	GAACTTCAGGGTCAGCTTGC	60
